# ﻿New descriptions and new records of the braconid parasitoids subfamilies Doryctinae and Rhyssalinae (Hymenoptera, Braconidae) in the fauna of South Korea

**DOI:** 10.3897/zookeys.1138.94580

**Published:** 2023-01-05

**Authors:** Sergey A. Belokobylskij, Deokseo Ku

**Affiliations:** 1 Zoological Institute, Russian Academy of Sciences, St Petersburg 199034, Russia Zoological Institute, Russian Academy of Sciences St Petersburg Russia; 2 The Science Museum of Natural Enemies, Geochang 50147, Republic of Korea The Science Museum of Natural Enemies Geochang Republic of Korea

**Keywords:** Descriptions, diagnoses, Ichneumonoidea, new records, new species, parasitoid

## Abstract

Five doryctine species, *Aivalykuskseniae***sp. nov.**, *Dendrosotinusgajwadongus***sp. nov.**, Doryctes (Plyctes) jinjuensis**sp. nov.**, Neoheterospilus (Neoheterospilus) geochangus**sp. nov.**, and *Spathiusfumipennis***sp. nov.**, are described as new for sciences from South Korea. Five doryctine genera, *Aivalykus* Nixon, *Dendrosoter* Wesmael, *Dendrosotinus* Telenga, *Guaygata* Marsh and *Pareucorystes* Tobias, and fifteen species are recorded in the fauna of the Korean Peninsula for the first time. Additionally, two genera from the subfamily Rhyssalinae, *Proacrisis* Tobias and *Histeromerus* Wesmael, and two species, *Proacrisisorientalis* Tobias, 1983 and *Histeromerusorientalis* Chou & Chou, 1991, are recorded in the fauna of Korea for the first time.

## ﻿Introduction

The fauna of the parasitoid family Braconidae of the Eastern Palaearctic is considerably diverse and abundant with numerous taxa penetrating here from the Oriental region. Despite the number of publications dedicated to the braconid subfamily Doryctinae for this zoogeographic region (e.g., Belokobylskij 1996; Belokobylskij and Maetô 2009; Belokobylskij et al. 2013; Tang et al. 2013, 2015; Belokobylskij and Ku 2021, etc.), the number of new records and species for this group continues increasing every year.

Information about the Doryctinae from the Korean Peninsula has been published in several survey and faunistic articles (Papp 1984; Belokobylskij 1998; Ku et al. 2001; Lee et al. 2020; Belokobylskij and Ku 2021, etc.).

In this article, five new doryctine species are described from Korea, seven genera and 17 species from subfamilies Doryctinae and Rhyssalinae are recorded for the first time for the Korean Peninsula.

## ﻿Materials and methods

The terminology employed for the morphological features, sculpture, and body measurements follows Belokobylskij and Maetô (2009). The wing venation nomenclature follows Belokobylskij and Maetô (2009), with the terminology of van Achterberg (1993) shown in parentheses. The new distribution records presented in this paper are marked with an asterisk (*).

The specimens were examined using an Olympus SZ51 stereomicroscope. Photographs were taken with an Olympus OM-D E-M1 digital camera mounted on an Olympus SZX10 microscope (Zoological Institute of the Russian Academy of Sciences, St Petersburg, Russia). Image stacking was performed using Helicon Focus 8.0. The figures were produced using the Adobe Photoshop CS6 program.

The specimens examined in this study were deposited in the collections of the National Institute of Biological Resources (Incheon, Republic of Korea; **NIBR**), the Science Museum of Natural Enemies (Geochang, Republic of Korea; **SMNE**), and the Zoological Institute of the Russian Academy of Sciences (St Petersburg, Russia; **ZISP**).

## ﻿Taxonomic account

**Class Hexapoda Blainville, 1816**

**Order Hymenoptera Linnaeus, 1758**

**Family Braconidae Nees, 1811**

### ﻿Subfamily Doryctinae Foerster, 1863

#### Species from the Korean Peninsula new for science

##### ﻿Genus Aivalykus

Taxon classificationAnimaliaHymenopteraBraconidae

Nixon, 1938

D2213D81-F0CD-5945-BB9A-1E6168C93600

###### Type species.

*Aivalykuseclectus* Nixon, 1938.

###### Notes.

The genus *Aivalykus* Nixon from the tribe Ecphylini is recorded in the fauna of Korea for the first time. This genus is unknown yet in southern regions of the Russian Far East and Japan (Belokobylskij 1996, 1998; Belokobylskij and Maetô 2009), though it has been found in the south of China (Belokobylskij and Chen 2002).

##### ﻿ Aivalykus kseniae sp. nov.

Taxon classificationAnimaliaHymenopteraBraconidae

389B1B2A-B126-545A-9EEE-ADA2C429E17C

https://zoobank.org/659BD181-E950-4827-BAB4-3D11FD877281

Figs 1 , 2

###### Type material.

***Holotype***: female, “Korea (GB), Ian yeomul san [Ian-myeon, Yeomul-ri San] 39–4, Sangji-[shi], 36°32'46.9"N, 128°07'46.6"E, 2020.V.24–VI.12, Coll. S.S. Kim, The 5^th^ National Ecosystem Survey” (NIBR).

***Paratype***: 1 female, “Korea (GB), Cheonbu3-gil, Buk-myeon, Ulleung-gun, V.23–VI.7.2017 (Malaise trap), Ku Deokseo” (SMNE).

###### Comparative diagnosis.

This new species is similar to *Aivalykusnitidus* Belokobylskij & Chen, 2002 (Belokobylskij and Chen 2002), but differs by having the vertex with a distinct aciculation (very finely aciculate in *A.nitidus*), five carinae on the prescutellar depression (only a single median carina in *A.nitidus*), second medial abscissa (2-SR+M) short, recurrent vein (m-cu) weakly antefurcal, ~ 8.0× longer than second medial abscissa (2-SR+M) (long, strongly antefurcal, in 1.6–2.0× longer in *A.nitidus*), brachial cell closed weakly before recurrent vein (distinctly before it in *A.nitidus*), setae on the dorsal margin of the hind tibia short, 0.3–0.5× as long as the maximum width of the tibia (long, 0.7–0.8× as long as the width of the tibia in *A.nitidus*), and apical segments of antenna dark brown to black (three apical segments white in *A.nitidus*).

###### Description.

**Female.** Body length 2.2–2.5 mm; fore wing length 2.0–2.2 mm; ovipositor sheath length 1.7–2.0 mm.

***Head*.** Head width (dorsal view) 1.5–1.6× its median length, 1.1× width of mesoscutum. Head behind eye (dorsal view) weakly convex or subparallel in anterior 1/2, roundly narrowed in posterior 1/2; transverse diameter of eye 1.6–1.8× length of temple. Ocelli small, arranged in equilateral triangle with base 1.1–1.2× its sides. POL 1.1–1.2× Od, 0.5–0.6× OOL. Eye glabrous, 1.1–1.2× as high as broad. Malar space 0.4–0.5× height of eye, almost equal to basal width of mandible. Face width 0.9–1.0× height of eye and almost equal to height of face and clypeus combined. Malar suture absent. Clypeus high, 1.0–1.2× as wide as high. Clypeal suture shallow, distinct laterally and almost absent upper medially. Hypoclypeal depression round, its transverse width 0.4–0.5× distance from edge of depression to eye, 0.3–0.4× width of face. Occipital carina reduced below, not fused with hypostomal carina.

***Antenna*.** Antenna slender, almost filiform, 18–21-segmented, weakly longer than body. Scape 1.2× longer than its maximum width. First segment of flagellum not flattened, weakly curved, 3.8–4.0× longer than its apical width, 0.7–0.8× longer than second segment. Penultimate segment 4.0–4.5× longer than wide, 0.8–0.9× as long as first segment, as long as apical segment; the latter almost obtuse apically.

***Mesosoma*.** Mesosoma 1.7–1.8× longer than high. Neck of prothorax short. Pronotal carina distinct. Mesoscutum highly and almost perpendicularly elevated above pronotum (lateral view), ~ 1.1× wider than its medial length (dorsal view). Notauli deep in anterior 1/2, shallow in posterior 1/2, anteriorly distinctly crenulate. Prescutellar depression (scutal sulcus) deep, with five complete or sometimes partly incomplete longitudinal carinae, smooth between carinae, 0.3× as long as weakly convex scutellum. Subalar depression shallow, wide, distinctly obliquely striate. Precoxal sulcus very shallow and narrow, finely longitudinally aciculate or smooth, connected with prepectal carina anteriorly, running along ~ 1/2 of lower part of mesopleuron. Metanotum almost without tooth.

***Wings*.** Fore wing 3.0–3.3× longer than its maximum width. Radial vein (r) arising almost from middle of pterostigma. Radial (marginal) cell weakly shortened. Metacarp (1-R1) almost as long as pterostigma. First radial abscissa (r) perpendicular to pterostigma, 0.7–0.9× as long as maximum width of pterostigma, 0.5–0.6× as long as first radiomedial vein (2-SR). Second radial abscissa (3-SR+SR1) distinctly evenly curved, 6.6–7.3× longer than first abscissa (r), 3.8–3.9× longer than first radiomedial vein (2-SR). Discoidal (first discal) cell ~ 2.0× longer than wide. Recurrent vein (m-cu) weakly antefurcal, 4.0–6.0× longer than second abscissa of medial vein (2-SR+M), 0.6–0.7× as long as first radiomedial vein (2-SR). Brachial (first subdiscal) cell narrow, gently closing apically weakly before recurrent vein (m-cu). Distance from nervulus (cu-a) to basal vein (1-M) 0.5–1.0× nervulus (cu-a) length. In hind wing medial (basal) cell closed antero-distally. Recurrent vein (m-cu) absent, or sometimes present, but short and strongly desclerotised.

**Figure 1.**
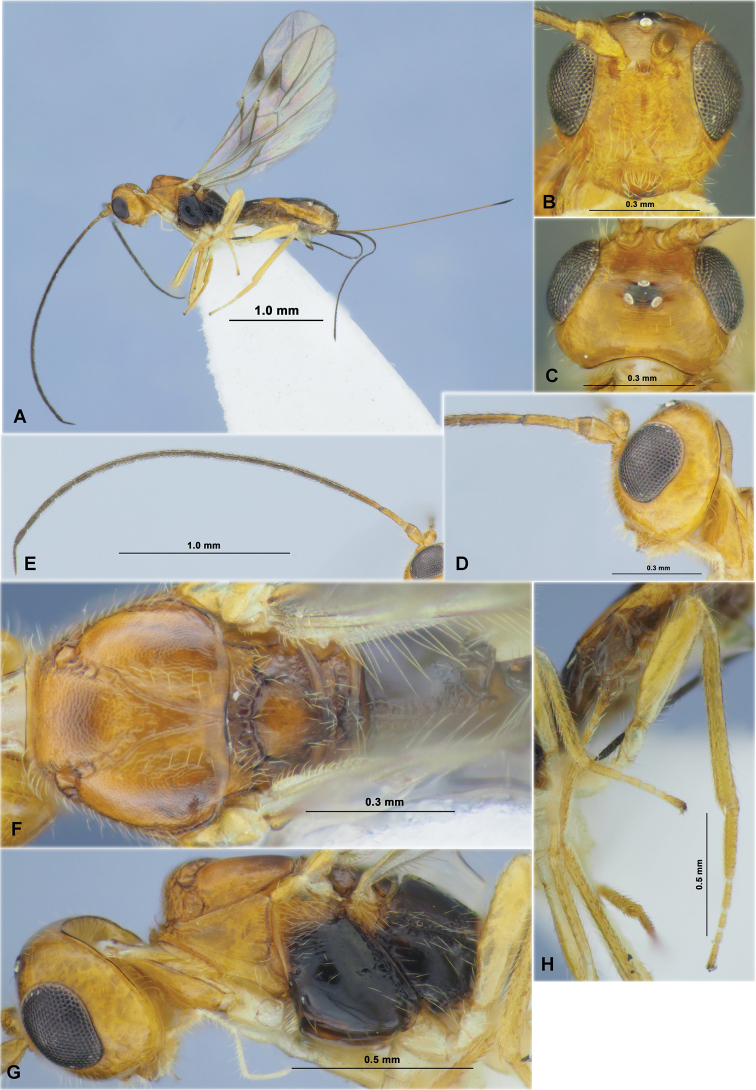
*Aivalykuskseniae* sp. nov. (female, holotype) **A** habitus, lateral view **B** head, front view **C** head, dorsal view **D** head, lateral view **E** antenna **F** mesosoma, dorsal view **G** head and mesosoma, lateral view **H** hind leg.

**Figure 2.**
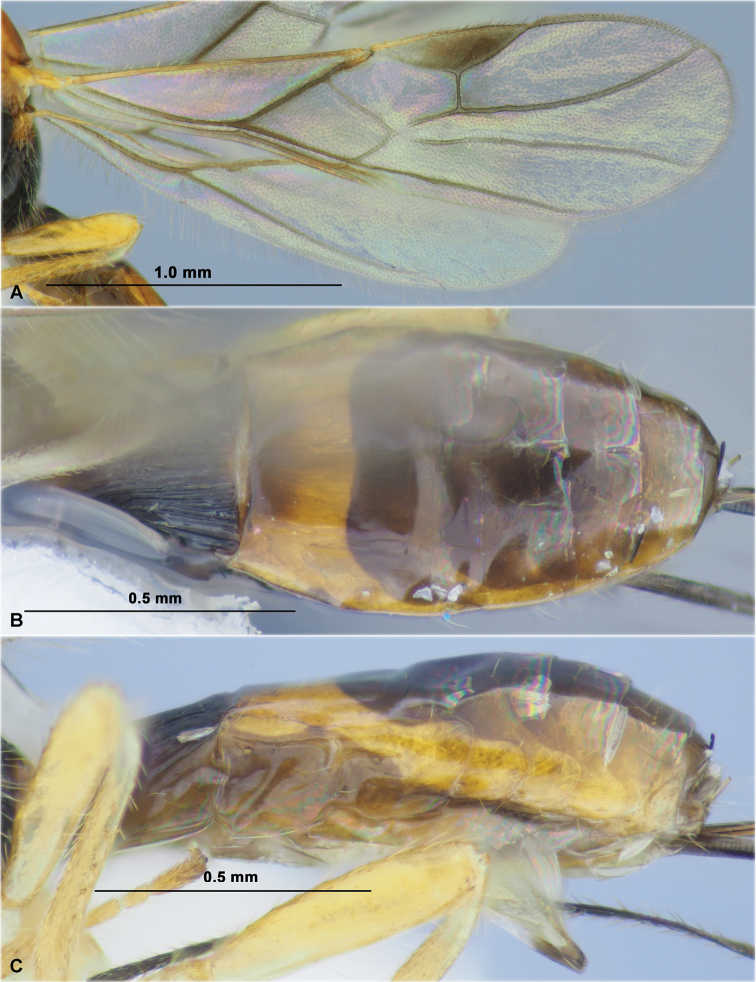
*Aivalykuskseniae* sp. nov. (female, holotype) **A** wings **B** metasoma, dorsal view **C** metasoma, lateral view.

***Legs*.** Hind femur 3.8–4.0× longer than wide. Hind tarsus 0.75–0.80× as long as hind tibia. Hind basitarsus thickened, thicker than following segments, 0.7–0.8× as long as second–fifth segments combined. Second segment 0.4× as long as basitarsus, 1.1–1.3× longer than fifth segments (without pretarsus).

***Metasoma*.** Metasoma 0.9–1.0× as long as head and mesosoma combined. First tergite without spiracular tubercles, spiracles situated on basal 1/3 of tergite, distinctly and linearly widened from base to apex. Maximum width of first tergite 1.7–2.0× its minimum width, length 1.10–1.15× its apical width, 1.3–1.5× length of propodeum. Second tergite without sublateral oblique depressions. Suture between second and third tergites indistinct. Medial length of second and third tergites combined 1.1–1.2× basal width of second tergite, 0.8× their maximum width. Ovipositor sheath 0.8× as long as body, 1.4–1.6× longer than metasoma, 2.0–2.2× longer than mesosoma, 0.8–0.9× as long as fore wing.

***Sculpture and pubescence*.** Vertex almost entirely aciculate; frons mainly smooth with fine aciculation posteriorly or widely and finely aciculate; temple smooth; face mainly smooth with sparse punctation, finely aciculate submedially on narrow stripes and below. Sides of pronotum mainly smooth but striate marginally. Mesoscutum distinctly and densely coriaceous, sometimes sculpture situated in irregular transverse dense striae anteriorly; with two middle and strongly convergent posteriorly longitudinal carina in posterior 1/2. Scutellum almost entirely smooth. but finely coriaceous laterally. Mesopleuron and metapleuron mainly smooth. Propodeum mainly smooth, with coarse and short rugulosity along median carinae in basal 2/3, with distinctly delineated by carinae, short and relatively wide smooth areola in posterior 1/3 of propodeum. Hind coxa and femur smooth. First metasomal tergite with distinct, complete, and closely situated dorsal carinae, entirely densely and distinctly striate. Remaining tergites completely smooth. Hind tibia on dorsal surface with rather sparse and semi-erect pale setae, length of these setae 0.3–0.5× maximum width of hind tibia.

***Colour*.** Head and anterior 1/2 of mesosoma pale reddish brown to yellowish brown; posterior 1/2 of mesosoma and first metasomal tergite dark brown to black, remaining part of metasoma reddish brown with yellowish margins. Antenna dark brown to black (including subapical and apical segments), three basal segments yellowish brown. Palpi pale yellow. Legs brownish yellow or yellow. Ovipositor sheath black. Wings faintly infuscate; pterostigma brown, but pale yellow in its basal quarter.

**Male.** Unknown.

###### Etymology.

Named after the daughter of the first author, Ksenia.

###### Distribution.

Korean Peninsula.

##### ﻿Genus Dendrosotinus

Taxon classificationAnimaliaHymenopteraBraconidae

Telenga, 1941

9111BA76-F181-5E7D-8E01-600A0716840A

###### Type species.

*Dendrosoterferrugineus* Marshall, 1888.

###### Notes.

The genus *Dendrosotinus* Telenga, 1941 from the tribe Doryctini is recorded in the fauna of Korea for the first time.

##### ﻿ Dendrosotinus (Gildoria) gajwadongus sp. nov.

Taxon classificationAnimaliaHymenopteraBraconidae

C5DD49EE-9D11-572B-8055-57EB77A96201

https://zoobank.org/BA9A4A3F-5612-459E-8F90-EF755807AF90

Fig. 3

###### Type material.

***Holotype***: male, “S. Korea, Gyeongsangnam-do, Jinju-[shi], Gajwa-dong, V.1993, D.-S. Ku leg.”(NIBR)

###### Comparative diagnosis.

According to van Achterberg (2003), this species is similar to the briefly described Western Palaearctic Dendrosotinus (Gildoria) planus (Ratzeburg, 1848), but differs from the latter species by having the antenna 13-segmented (20-segmented in *D.planus*), brachial vein (CU1b) distinctly oblique to the mediocubital vein (2-CU1) (subperpendicular in *D.planus*), mesoscutum coarsely rugose-reticulate and without granulation (densely reticulate-granulate in *D.planus*), and second metasomal tergite longitudinally striate with reticulation (finely aciculate in *D.planus*).

###### Description.

**Male.** Body length 1.3 mm; fore wing length 1.1 mm.

***Head*.** Head width (dorsal view) 1.5× its median length, 1.2× width of mesoscutum. Head behind eyes (dorsal view) subparallel in anterior 1/2 and roundly narrowed in posterior 1/2. Transverse diameter of eye (dorsal view) 1.2× longer than temple. Ridge on border of vertex and frons absent. Ocelli small, arranged in triangle with base 1.3× its sides. POL 1.5× Od, ~ 0.7× OOL. Eye bare, almost without emargination opposite antennal sockets, 1.1× as high as broad. Malar suture absent. Malar space 0.4× height of eye, 0.8× basal width of mandible. Face width 1.1× height of eye and 1.3× height of face and clypeus combined. Clypeus with distinct short lower flange. Clypeal suture distinct. Hypoclypeal depression subround, its width 0.6× distance from edge of depression to eye, 0.35× width of face. Occipital carina complete dorsally, obliterated ventrally at rather long distance and not fused with hypostomal carina.

***Antenna*.** Antenna slender, filiform, 13-segmented, almost as long as body. Scape 1.6× longer than its maximum width, 1.4× longer than pedicel. First flagellar segment not widened, almost not curved, not convex and without sculpture on its outer side, weakly concave and smooth on inner side, ~ 5.0× longer than its maximum width, 0.8× as long as second segment. Penultimate segment 4.7× longer than wide, approximately as long as apical segment; the latter weakly acuminated.

**Figure 3.**
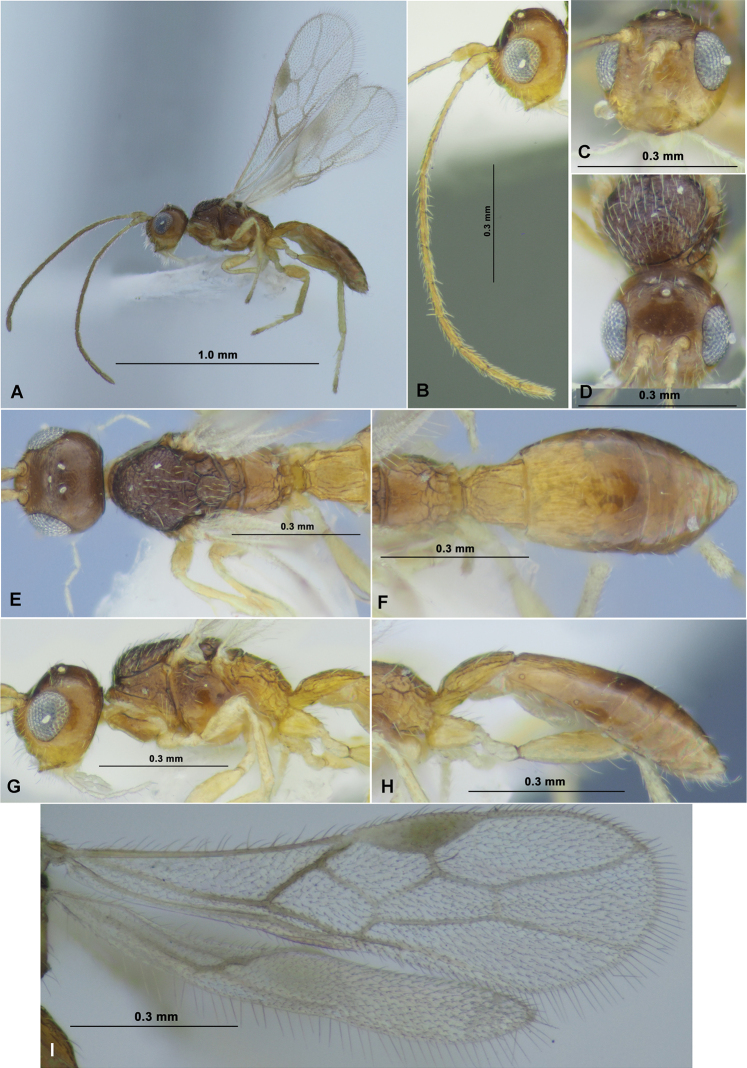
Dendrosotinus (Gildoria) gajwadongus sp. nov. (male, holotype) **A** habitus, lateral view **B** head and antenna, lateral view **C** head, front view **D** mesoscutum and head, dorso-anterior view **E** head, mesosoma and first metasomal tergite, dorsal view **F** propodeum and metasoma, dorsal view **G** head, mesosoma and first metasomal tergite, lateral view **H** propodeum and metasoma, lateral view **I** wings.

***Mesosoma*.** Mesosoma not depressed, its length 1.8× maximum height. Pronotum short, dorsally with weakly convex lobe, with distinct and high pronotal keel; side of pronotum with wide, shallow, and curved submedian furrow. Mesoscutum highly and convex-roundly elevated above pronotum (lateral view). Median lobe of mesoscutum distinctly protruding forwards. Notauli rather wide, deep anteriorly and shallow posteriorly, crenulate-rugulose. Prescutellar depression rather deep, relatively short, with four distinct carinae, finely rugulose between carinae, 0.3× as long as convex scutellum. Subalar depression shallow, wide, distinctly and widely rugose-reticulate. Precoxal sulcus distinct, relatively deep, straight, smooth, connected with prepectal carina anteriorly, running along anterior 1/2 of lower part of mesopleuron. Propodeum without lateral tubercles.

***Wings*.** Fore wing 3.3× longer than its maximum width. Pterostigma 3.3× longer than wide. Radial vein (r) arising almost from middle of pterostigma. Radial (marginal) cell weakly shortened. Metacarp (1-R1) inside of radial (marginal) cell almost as long as pterostigma. Second radial abscissa (3-SR) 3.0× longer than first abscissa (r), 0.4× as long as weakly curved third abscissa (SR1), as long as first radiomedial vein (2-SR). Second radiomedial (submarginal) cell medium-sized, not narrowed distally, 2.6× longer than its maximum width, 1.7× longer than narrow brachial (subdiscal) cell. Recurrent vein (m-cu) distinctly postfurcal, 1.5× longer than second abscissa of medial vein (2-SR+M), 0.4× as long as first radiomedial vein (2-SR). Distance (1-CU1) between nervulus (cu-a) to basal vein (1-M) almost equal to nervulus (cu-a) length; nervulus (cu-a) straight and perpendicular to mediocubital vein (M+CU1). Parallel vein (CU1a) interstitial. Brachial (subdiscal) cell distally closed distinctly before recurrent vein (m-cu); apical vein of longitudinal anal vein (2A+3A) behind brachial vein absent. Hind wing with three hamuli, ~ 7.0× longer than wide. First abscissa of costal vein (C+SC+R) 0.6× as long as second abscissa (1-SC+R). First abscissa of mediocubital vein (M+CU) 0.6× as long as second abscissa (1-M). Recurrent vein (m-cu) mainly unsclerotised, oblique toward apex of wing, strongly antefurcal.

***Legs*.** Fore tibia with fine spines arranged in almost single row. Hind coxa practically without basoventral tubercle. Hind femur without dorsal protuberance, ~ 3.0× longer than wide. Hind tarsus 0.9× as long as hind tibia. Basitarsus widened distally, with long ventral thorn on its inner corner, 0.45× as long as second–fifth segments combined. Second tarsal segment 0.4× as long as basitarsus, 0.5× as long as fifth segment (without pretarsus).

***Metasoma***. Metasoma almost as long as head and mesosoma combined. First tergite with small dorsope, with distinct spiracular tubercles in its basal 1/3; tergite distinctly and almost linearly widened from base to basal 1/3, then very weakly and sublinearly widened towards apex. Maximum width of first tergite 1.8× its minimum width; length of tergite 1.2× its apical width. Second suture rather distinct, shallow, weakly curved and without sublateral breaks. Second tergite 0.8× as long as its basal width, 1.2× longer than third tergite. Medial length of second and third tergites combined 1.4× basal width of second tergite, almost as long as their maximum width.

***Sculpture and pubescence*.** Vertex and frons weakly and rather sparsely reticulate-coriaceous; face densely transversely striate laterally and smooth medially; temple smooth. Mesoscutum entirely distinctly and densely rugulose-reticulate, without additional granulation. Scutellum very finely and densely reticulate-granulate. Mesopleuron smooth in lower 1/2, reticulate-coriaceous in upper 1/2. Propodeum with areas delineated by distinct carinae; basolateral areas mainly reticulate-coriaceous but anteriorly partly smooth; areola long and narrow, with several transverse carinae, ~ 2.5× longer than maximum with, petiolate areas not delineated; basal carina short, 0.4× as long as anterior fork of areola. Hind coxae mainly smooth, but curvedly striate dorsally. Hind femur entirely smooth. First metasomal tergite entirely and sparsely curvedly striate, with distinct and dense reticulation between striae; second tergite entirely weakly longitudinally striate with reticulation. Remaining tergites smooth. Vertex widely glabrous medially, anteriorly, and laterally with sparse, short, and semi-erect white setae. Mesoscutum entirely with sparse, short, and white semi-erect setae. Metapleuron medially widely glabrous. Hind tibia dorsally with sparse, short, and semi-erect setae, its length 0.8–1.0× maximum width of hind tibia.

***Colour*.** Head reddish brown, distinctly infuscate dorsally, brownish yellow ventrally. Mesosoma reddish brown, dark reddish brown on mesoscutum and scutellum, prosternum and propodeum brownish yellow. Metasoma brownish yellow in basal 1/2, reddish brown with dark transverse stripes on posterior margin of tergites in apical 1/2. Antenna brown, three basal segments yellow. Palpi pale yellow. Legs brownish yellow to yellow. Fore wing hyaline; pterostigma mainly brown, yellow basally.

**Female.** Unknown.

###### Etymology.

Named after the type locality of the new species in South Korea, Gajwa-dong, Jinju City.

###### Distribution.

Korean Peninsula.

###### Remarks.

Despite the intensive study for the braconid fauna of the Korean Peninsula in the last period, only a single male of this species has been collected till now. However, the distinct diagnostic characters of this new species allow us to easily separate it from remaining described Asian species of *Dendrosotinus*.

A species of *Dendrosotinus* described in Chinese from Fujian Province (China), *D.wuyiensis* Shi, 2006 (Shi 2006), perhaps belongs to the genera *Ontsira* Cameron, 1900 or *Neurocrassus* Snoflak, 1945 according to the figures provided in the original description. However, only a study of the holotype of this species will allow to confirm its real taxonomic position.

### ﻿Key to the Asian species of the genus *Dendrosotinus*

**Table:** 

1	Third antennal segment (especially of female) widened, more or less depressed and anteriorly sculptured. Recurrent vein (m-cu) of fore wing subinterstitial; brachial (subdiscal) cell moderately wide. (Subgenus Dendrosotinus s. str.). – Armenia, Azerbaijan, former Yugoslavia, France, Greece, Israel, Italy, Russia (North Caucasus), Saudi Arabia, Spain, Turkey, UAE	**D. (D.) ferrugineus (Marshall, 1888)**
–	Third antennal segment slender, cylindrical, and anteriorly usually smooth. Recurrent vein (m-cu) of fore wing distinctly postfurcal; brachial (subdiscal) cell narrow (subgenus Gildoria Hedqvist, 1974)	**2**
2	Brachial (subdiscal) cell of fore wing closed distinctly before recurrent vein (m-cu)	**3**
–	Brachial (subdiscal) cell of fore wing closed on or weakly before or behind of recurrent vein (m-cu)	**5**
3	Antenna 13-segmented (Fig. 3B). Vertex very weakly coriaceous (Fig. 3D, E). Fore wing entirely hyaline (Fig. 3I). Mesoscutum coarsely rugose-reticulate and without granulation (Fig. 3D, E). – Basitarsus of hind tarsus with long ventral thorn on its inner corner (Fig. 3A). Body length 1.3 mm. – Korean Peninsula	***D. (G.) gajwadongus* sp. nov.**
–	Antenna 18–22-segmented. Vertex distinctly densely reticulate-areolate and with granulation. Fore wing faintly maculate, with rather distinctly infuscate transverse stripes. Mesoscutum transverse striate-rugose, and with additional granulation	**4**
4	Transverse diameter of eye (dorsal view) 1.9–2.3× longer than temple. Malar space 0.4× maximum diameter of eye. Length of first tergite 1.1–1.2× its maximum posterior width. Second tergite without sublateral depression. Ovipositor sheath 0.5–0.6× as long as metasoma, 0.4–0.5× as long as fore wing. Body mainly pale reddish brown or reddish brown, darkened dorsally. Body length 2.2–2.8 mm. – Yemen	**D. (G.) maculipennis Belokobylskij, 2021**
–	Transverse diameter of eye (dorsal view) 1.2–1.4× longer than temple. Malar space 0.6× maximum diameter of eye. Length of first tergite 1.3–1.4× its maximum posterior width. Second tergite with very shallow, subparallel, sublateral and almost straight depression in anterior 1/2. Ovipositor sheath 0.9–1.0× as long as metasoma, 0.7–0.8× as long as fore wing. Body mainly brownish yellow to partly yellow. Body length 2.0–2.5 mm. – UAE	**D. (G.) subelongatus Belokobylskij, 2021**
5	Transverse diameter of eye 1.2–1.3× longer than temple (dorsal view). Parallel vein (CU1a) interstitial to mediocubital vein (2-CU1). First metasomal tergite as long as its apical width. Second metasomal tergite weakly rugulose-reticulate in medio-basal 1/2. Body length 3.3 mm. – Tajikistan	***D.anthaxiae* Belokobylskij, 1983**
–	Transverse diameter of eye 2.0–2.2× longer than temple (dorsal view). Parallel vein (CU1a) not interstitial to mediocubital vein (2-CU1), arising from anterior fourth of the vein (3-CU1) closing brachial (subdiscal) cell apically. First metasomal tergite 1.2–1.3× longer than apical width. Second metasomal tergite entirely striate with reticulation	**6**
6	Vertex distinctly and densely transverse striate with fine reticulation between striae. Antenna 36-segmented. Hind femur 3.0× longer than wide. Third metasomal tergite sculptured baso-laterally. Ovipositor sheath 1.3× longer than metasoma. Body length 3.3 mm. – China (Taiwan)	***D.taiwanicus* Belokobylskij, 2010**
–	Vertex weakly and densely granulate, without striation. Antenna 27-segmented. Hind femur 2.3× longer than wide. Third metasomal tergite entirely smooth. Ovipositor sheath 0.4× as long as metasoma. Body length 3.3 mm. – Vietnam	***D.gratus* Belokobylskij, 1993**

#### ﻿Genus Doryctes

Taxon classificationAnimaliaHymenopteraBraconidae

Haliday, 1836

AD9EC5D6-9679-55DA-91A5-4764D00E7DD6

##### Type species.

*Braconobliteratus* Nees, 1834.

###### Subgenus Plyctes Fischer, 1981

#### ﻿ Doryctes (Plyctes) jinjuensis sp. nov.

Taxon classificationAnimaliaHymenopteraBraconidae

19E26E94-06EE-5B77-B16C-F11E53B76DDA

https://zoobank.org/70CA97A4-2D2D-4394-8529-91846C7BE301

Figs 4 , 5 , 6

##### Type material.

***Holotype***: female, “S. Korea: Gyeongsangnam-do, Sancheong-gun, [Chahwang-myeon], 30 km NNW Jinju (Chinju), forest, bush, h = 800 m, 29.06.2002, S. Belokobylskij” (NIBR).

***Paratypes*.** 1 male, “Korea, Gyeongnam-do, Jinju-si [=shi], Gajwa-dong, 27. X.–3.XI.1987, Malaise trap. D-S Ku” (SMNE); 1 male, “Korea, Gyeongnam-do, Jinju-si [=shi], Gajwa-dong, 15.–21.VII.1989. Malaise trap (Black). D-S Ku” (ZISP); 1 male, “Korea, Gyeongbuk-do, Gyeongsan-si [=shi], Yeungnam University, 12.VIII.1987, J-Y Cha” (SMNE); 1 male, “Korea: KK [=GG], Suwon, Mt. Yeogi, MT (B1/B1), 8.IX.1997, June-Yeol Choi” (SMNE).

##### Comparative diagnosis.

This new species is very similar to Doryctes (Plyctes) diversus (Szépligeti, 1910) and D. (P.) malayensis Fullaway, 1919; the differences between these species are given in the key below.

##### Description.

**Female.** Body length 6.5 mm; fore wing length 5.0 mm.

***Head*.** Head width (dorsal view) 1.3× its median length, 1.15× maximum width of mesoscutum. Head behind eyes (dorsal view) weakly convex in anterior 1/2, roundly narrowed in posterior 1/2. Transverse diameter of eye 1.4× longer than temple (dorsal view). Ocelli medium-sized, arranged in triangle with base 1.2–1.3× its side. POL 1.3× OD, 0.6× OOL. Eye glabrous, with very weak emargination opposite of antennal socket, 1.15× as high as broad. Malar space 0.3× height of eye, 0.6× as long as basal width of mandible. Malar suture very shallow. Face width ~ 0.8× height of eye, 1.2× height of face and clypeus combined. Hypoclypeal cavity round, its diameter equal to distance from margin of cavity to border of eye, 0.5× as long as width of face. Occipital carina complete dorsally, obliterated below at rather long distance and not fused with hypostomal carina.

***Antenna*.** Antenna slender, setiform, more than 43-segmented (apical segments missing). Scape 1.9× longer than its maximum width. First flagellar segment ~ 5.0× longer than its apical width, 1.05× longer than second segment. Subapical segment 4.7× longer than its maximum width.

***Mesosoma*.** Length of mesosoma 2.1× longer than its height. Pronotum dorsally with weakly convex dorsal lobe (lateral view) and with distinct pronotal keel in anterior 1/3. Mesoscutum distinctly and curvedly elevated above pronotum. Median lobe of mesoscutum anteriorly distinctly convex and protruding forwards (dorsal view). Notauli anteriorly deep, wide and crenulate, posteriorly very shallow, narrow, and smooth, almost complete. Prescutellar depression rather deep, almost entirely distinctly rugose, with median carina, 0.4× as long as scutellum. Scutellum weakly convex, with weak lateral carinae. Subalar depression shallow, rather wide, weakly striate-rugose. Precoxal sulcus deep, long, smooth, without round cavity medially or posteriorly, running along anterior 2/3 of lower part of mesopleuron. Propodeum without lateral tubercles.

**Figure 4.**
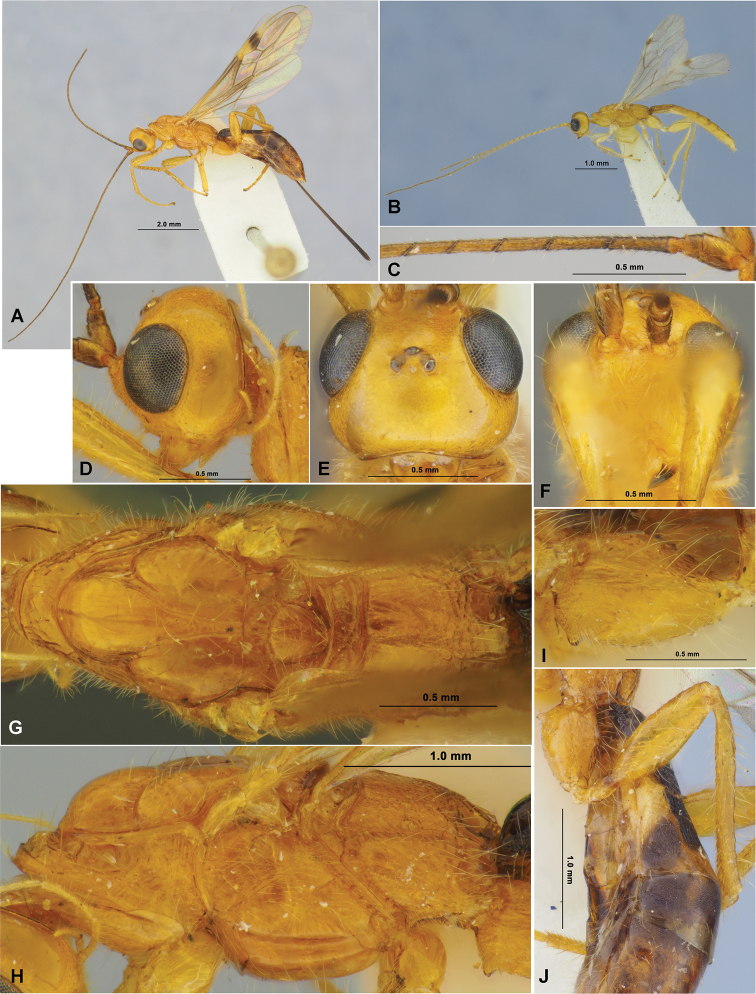
Doryctes (Plyctes) jinjuensis sp. nov. (female, holotype **A, C–J** male, paratype **B**) **A, B** habitus, lateral view **C** basal segments of antenna **D** head, lateral view **E** head, dorsal view **F** head, front view **G** mesosoma, dorsal view **H** mesosoma, lateral view **I** hind coxa, lateral view **J** hind leg.

**Figure 5.**
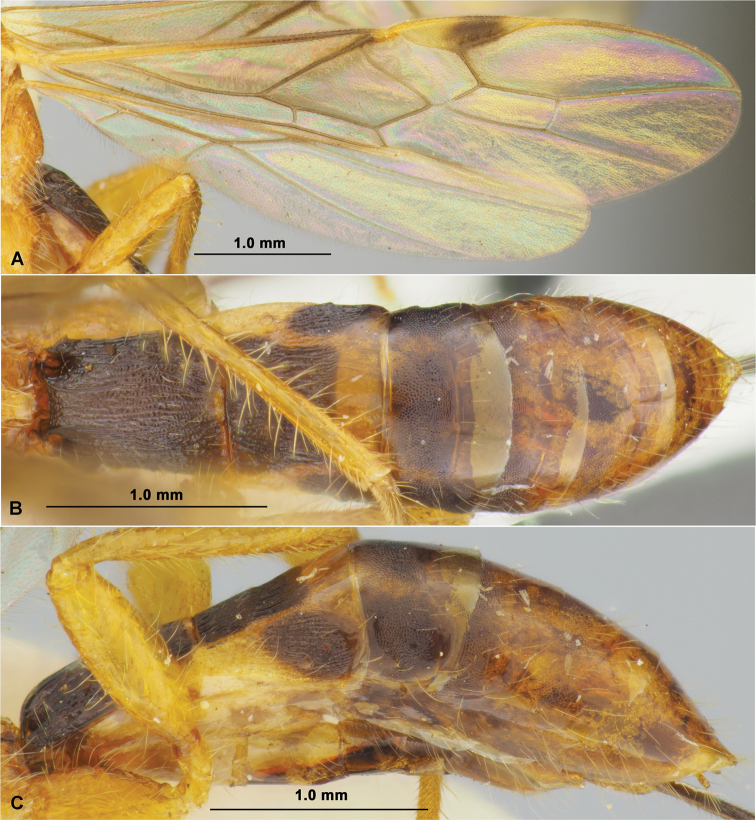
Doryctes (Plyctes) jinjuensis sp. nov. (female, holotype) **A** wings **B** metasoma, dorsal view **C** metasoma, lateral view.

**Figure 6.**
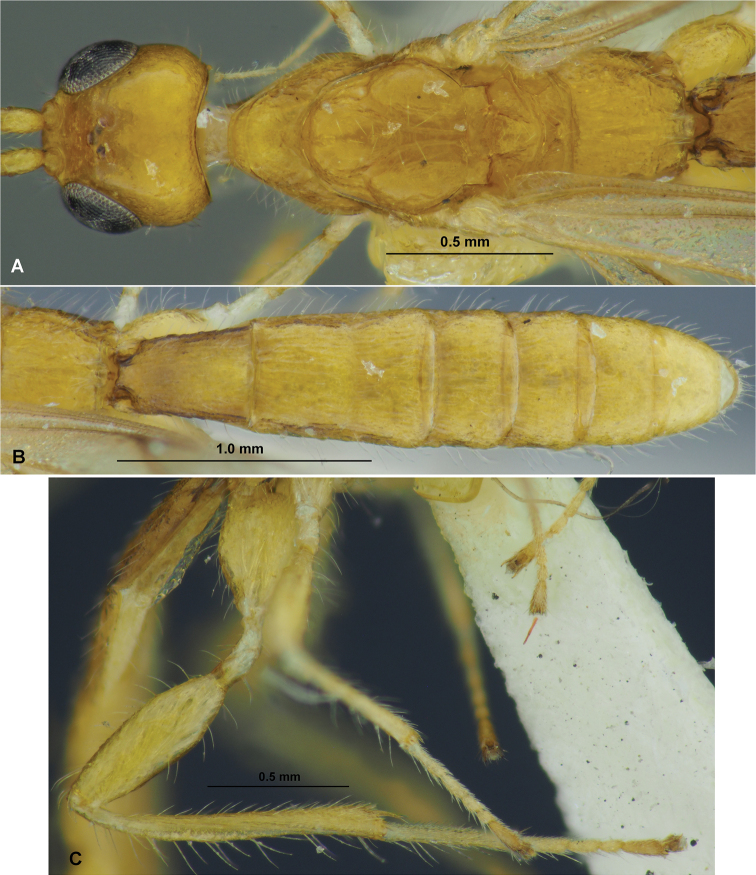
Doryctes (Plyctes) jinjuensis sp. nov. (male, paratype) **A** head and mesosoma, dorsal view **B** metasoma, dorsal view **C** hind leg, lateral view.

***Wings*.** Fore wing 3.6× longer than its maximum width. Pterostigma 4.3× longer than maximum width. Radial vein (r) arising almost from middle of pterostigma. Second radial abscissa (3-SR) forming very obtuse angle with first radial abscissa (r) and twice longer than it, 0.4× as long as third radial abscissa (SR1), 1.8× longer than first radiomedial vein (3-SR). Second radiomedial (submarginal) cell relatively short and narrow, 3.0× longer than its maximum width, 1.2× longer than wide brachial (first subdiscal) cell. First medial abscissa (1-SR+M) distinctly sinuate. Recurrent vein (m-cu) distinctly antefurcal, 5.5× longer than second medial abscissa (2-SR+M), 1.3× longer than first radiomedial vein (3-SR). Distance (1-CU1) between nervulus (cu-a) and basal vein (1-M) 1.5× nervulus (cu-a) length. Parallel vein (CU1a) arising from posterior 1/3 of distal margin of brachial (first subdiscal) cell. Hind wing 5.0× longer than maximum width. First costal abscissa (C+SC+R) 0.6× as long as second abscissa (1-SC+R). First abscissa of mediocubital vein (M+CU) almost equal to second abscissa (1-M). Recurrent vein (m-cu) entirely straight, oblique, weakly antefurcal.

***Legs*.** Hind coxa with low and wide dorsal protuberance, with distinct basoventral tubercle. Hind femur 3.0× longer than its maximum width. Hind tarsus almost as long as hind tibia. Hind basitarsus 0.8× as long as second–fifth segments combined; second segment of hind tarsus 0.4× as long as basitarsus, 1.3× longer than fifth segment (without pretarsus).

***Metasoma*.** Metasoma 1.3× longer than mesosoma and head combined. First tergite without distinct spiracular tubercles, weakly and almost linearly widened from subbase to apex. Maximum width of first tergite 1.7× its minimum basal width; its length 1.2× maximum subapical width. Second tergite with fine, almost straight, and subparallel sublateral furrows; median length of second tergite 0.45× its basal width, 0.8× length of third tergite. Suture between second and third tergites present, shallow, wide, and weakly concave medially, with not deep sublateral bends. Third tergite without depression. Ovipositor sheath almost as long as metasoma, 1.5× longer than mesosoma and 0.65× as long as fore wing.

***Sculpture and pubescence***. Vertex, frons, and temple smooth; face medially widely rugulose-striate, sparsely punctate to smooth laterally. Mesoscutum and scutellum smooth, with weak and short transverse striation between notauli in posterior 1/3 of mesoscutum. Mesopleuron mainly smooth. Propodeum with areas delineated by relatively weak carinae; basolateral areas long, smooth medially, reticulate-rugulose along carinae; areola rather long and narrow, densely rugose-reticulate with transverse striation, almost twice longer than its maximum width; petiolate area not delineated; basal carina relatively short, 0.3× as long as propodeum. Hind coxae mainly rugose-reticulate, weakly reticulate-coriaceous laterally; hind femur finely coriaceous to smooth. First and second metasomal tergites entirely and third in basal 1/2 densely striate with dense reticulation between striae; basal halves of second–seventh tergites very densely granulate-reticulate, with fine transverse striations posteriorly becoming weaker to posterior tergites; distal halves of third to seventh tergites smooth. Vertex widely glabrous medially, posteriorly, and laterally with sparse, long, curved and almost erect yellow setae. Mesoscutum mainly glabrous, with rather sparse, long, curved and erect to semi-erect yellow setae along notauli and laterally. Metapleuron medially widely glabrous. Hind tibia dorsally with rather sparse, long, and erect yellow setae, its length 0.8–1.2× maximum width of hind tibia.

***Colour*.** Head and mesosoma yellow to brownish yellow; metasoma dark reddish brown in basal 2/3 and reddish brown in apical 1/3, with lateral yellow spots on second tergite. Antenna dark brown, scape reddish brown dorsally. Palpi yellow. Legs mainly yellow, hind coxa brownish yellow, hind tibia yellow basally, similar colour as remainder parts of tibia. Wings very faintly infuscate. Pterostigma dark brown in medioposterior 1/3, yellow in basal 1/2 and apical fifth.

**Male.** Body length 4.2–5.3 mm; fore wing length 2.8–3.7 mm. POL 1.0–1.3× OD, 0.4–0.7× OOL. Rarely vertex anteriorly with very fine aciculation on short area. Antenna at least 35-segmented. Scape 1.4–1.5× longer than its maximum width. Penultimate segment 4.0× longer than wide; 0.8× as long as apical segment; the latter subacuminate apically. Mesosoma 2.2–2.4 (rarely almost 2.8) × longer than its height. Notauli posteriorly shallow but distinct; rarely area here with additional oblique striation. Prescutellar depression sometimes almost entirely smooth, usually weakly rugulose. Scutellum sometimes finely striate with striae curved posteriorly, but usually mainly smooth. Basolateral areas widely reticulate-rugulose, almost smooth only basally; areola indistinctly or distinctly delineated; basal carina ~ 0.4× as long as propodeum. Fore wing 3.9–4.3× longer than its maximum width. Pterostigma 4.0–4.3× longer than maximum width. Second radial abscissa (3-SR) 2.3–2.6× longer than first radial abscissa (r), 1.7–1.9× longer than first radiomedial vein (3-SR). Second radiomedial (submarginal) cell 2.7–3.3× longer than its maximum width, almost as long as brachial (first subdiscal) cell. Recurrent vein (m-cu) 2.5–3.3× longer than second medial abscissa (2-SR+M), almost as long as first radiomedial vein (3-SR). Hind wing 5.6–6.5× longer than maximum width. First abscissa of mediocubital vein (M+CU) 0.85–0.90× as long as second abscissa (1-M). Hind femur 3.2× longer than maximum width. Metasoma narrow, 1.1–1.2× longer than mesosoma and head combined. First tergite 1.5–1.6× longer than maximum subapical width; maximum width of first tergite 1.8–2.0× its minimum basal width. Second tergite with very fine and weakly divergent posteriorly sublateral furrows; median length of second tergite 0.8–1.0× its basal width, 1.1–1.3× length of third tergite. Basal 2/3–4/5 of third to sixth tergites densely longitudinally striate, densely granulate-reticulate between striae, their apical parts smooth. Colour. Body entirely or almost entirely yellow to brownish yellow, often first tergite distinctly infuscate at least laterally and basally. Antenna brown, yellow to brownish yellow in basal 1/3. Legs entirely yellow. Wings hyaline.

##### Etymology.

Named after the type locality of the new species in South Korea, Jinju City, in the environment of which the holotype of the new species was collected.

##### Distribution.

Korean Peninsula.

### ﻿Key to the Asian species of the subgenus Plyctes Fischer, 1981

**Table:** 

1	Brachial vein (CU1b) of fore wing subvertical to second abscissa of longitudinal anal vein (2-1A). Pterostigma always brown apically. Mesoscutum entirely with short, dense and semi-erect setae, without glabrous areas on lobes. Third tergite without wide subbasal transverse depression	**Subgenus Doryctes s. str.**
–	Brachial vein (CU1b) of fore wing distinctly slanted towards base of wing. Pterostigma pale brown or yellow apically. Mesoscutum with sparse, long, and semi-erect or erect setae along notauli and marginally, rather widely glabrous medially on all its lobes. Third tergite usually with wide crenulate subbasal transverse depression	**2**
2	First abscissa of mediocubital vein (M+CU) of hind wing distinctly shorter than its second abscissa (1-M)	**Subgenus Neodoryctes Szepligeti, 1914**
–	First abscissa of mediocubital vein (M+CU) of hind wing equal to or longer than its second abscissa (1-M) (Subgenus Plyctes Fischer, 1981)	**3**
3	First tergite short, its length 0.9–1.0× maximum width. Median length of second tergite ~ 0.3× its basal width. Ovipositor sheath shorter, 0.7–0.8× as long as metasoma and 0.30–0.45× as long as fore wing	**4**
–	First tergite long, its length 1.15–1.40× maximum width. Median length of second tergite 0.4–0.5× its basal width. Ovipositor sheath longer, 0.9–1.2× as long as metasoma and 0.6–0.9× as long as fore wing	**6**
4	Face almost entirely smooth. Body length 5.8–6.4 mm. – China (Hainan)	**D. (P.) hainanensis Belokobylskij, Tang, He & Chen, 2012**
–	Face medially widely and distinctly subtransversely striate and often with fine rugulosity or punctation between striae	**5**
5	Mesoscutum almost entirely or mainly granulate-coriaceous. Radial vein (r) arising before middle of pterostigma. Hind femur 2.7–3.0× longer than its maximum width. Hind tibia weakly thickened. Propodeum with areas rather distinctly delineated by carinae. Sublateral bands of suture between second and third tergites less strongly expressed. Body length 2.2–4.5 mm. – Russia (Primorskiy kray), Korean Peninsula, Japan	***D* . (*P* .) *punctatus* Belokobylskij, 1984**
–	Mesoscutum medially smooth, its lateral lobes very finely granulate-coriaceous. Radial vein (r) arising from middle of pterostigma. Hind femur 2.6× longer than its maximum width. Hind tibia distinctly thickened. Propodeum without areas delineated by carinae. Sublateral bands of suture between second and third tergites strongly expressed. Body length 5.5 mm. – Sri Lanka	***D* . (*P* .) *solox* Enderlein, 1912**
6	Pterostigma entirely yellow. Temple longer, transverse diameter of eye 1.3× longer than temple (dorsal view). Hind tarsus 1.2× longer than hind tibia. Second segment of hind tarsus 1.6× longer than fifth segment (without pretarsus). First tergite long, its length 1.4× maximum width. – Setae on dorsal margin of hind tibia short, their length 0.8–1.0× maximum width of tibia. Body length 8.0 mm. – China (Yunnan)	**D. (P.) flavistigma Belokobylskij, Tang, He & Chen, 2012**
–	Pterostigma medially widely brown, yellow basally and apically. Temple shorter, transverse diameter of eye 1.4–1.6× longer than temple (dorsal view). Hind tarsus almost as long as hind tibia. Second segment of hind tarsus 1.1–1.3× longer than fifth segment (without pretarsus). First tergite short, its length 1.15–1.30× maximum width	**6**
6	Metasomal tergites behind third tergite entirely smooth. Body entirely yellow or brownish yellow. Hind femur entirely brownish yellow. Body length 9.8 mm. – India, Sierra Leone	**D. (P.) nigricornis (Kriechbaumer, 1894) [D. (P.) coxalis (Turner, 1922)**]
–	Metasomal tergites behind third tergite basally densely reticulate-coriaceous, usually on wide area. At least metasoma mainly reddish brown or dark brown. Hind femur often distinctly infuscate	**7**
7	Setae on dorsal margin of hind tibia longer, their length 1.4–1.8× maximum width of tibia. Mesosoma pale reddish brown with dark spots in its posterior part. Hind tibia basally rather distinctly dark. Body length 4.4–9.2 mm. – Japan, China, India, Vietnam, Malaysia, Indonesia	**D. (P.) malayensis (Fullaway, 1919)**
–	Setae on dorsal margin of hind tibia shorter, their length 0.8–1.2× maximum width of tibia. Mesosoma entirely yellow to brownish yellow or mostly reddish brown. Hind tibia basally yellow or brownish yellow	**8**
8	Hind coxa with low and wide dorsal protuberance (Fig. 4I). Pterostigma of fore wing widely yellow in basal 1/3 (Fig. 5A). Recurrent vein (m-cu) of hind wing curved toward base of wing (Fig. 5A). Mesosoma entirely yellow to brownish yellow (Fig. 4A). Hind leg entirely yellow (Fig. 4J). Body length 4.2–6.5 mm. – Korean Peninsula	**D. (P.) jinjuensis sp. nov.**
–	Hind coxa without dorsal protuberance. Pterostigma of fore wing narrowly yellow in basal quarter only. Recurrent vein (m-cu) of hind wing curved toward apex of wing. Mesosoma anteriorly yellowish brown or reddish brown with dark spots, posteriorly distinctly infuscate on most part of propodeum and metapleuron. Hind leg mostly reddish brown to dark reddish brown. Body length 6.7–8.4 mm. – Indonesia	**D. (P.) diversus (Szépligeti, 1910)**

#### ﻿Genus Neoheterospilus

Taxon classificationAnimaliaHymenopteraBraconidae

Belokobylskij, 2006

878AFA26-9607-5171-A65B-BAF9C4073A98

##### Type species.

*Neoheterospiluskoreanus* Belokobylskij, 2006.

#### ﻿ Neoheterospilus (Neoheterospilus) geochangus sp. nov.

Taxon classificationAnimaliaHymenopteraBraconidae

CA02F855-D682-52F7-8A09-61A2145DEE83

https://zoobank.org/E181E56A-5066-4867-894C-18ECDBE40F79

Figs 7 , 8

##### Type material.

***Holotype***: 1 female, Korean Peninsula, “Korea (GN), Geochang-gun, Geochang-eup, Science Museum Natural Enemy, VI.3–VI.27.2015 (Malaise Trap), Ku Deokseo” (NIBR).

##### Comparative diagnosis.

This new species is similar to Neoheterospilus (N.) subtropicalis Belokobylskij, 2006 (Belokobylskij, 2006) from Japan, China, Korea and Vietnam, but differs from the latter species by having the 14-segmented slender antenna with first flagellar segment 6.5× longer than its apical width and 0.9× as long as second segment (thick and 16–17-segmented, with first flagellar segment 4.0–4.7× longer than its apical width and as long as second segment in *N.subtropicalis*), scape long, almost 2.0× longer than its maximum width (short, 1.3–1.5× in *N.subtropicalis*), precoxal sulcus running along almost the entire length of lower part of mesopleuron (only in anterior 1/2 in *N.subtropicalis*), basolateral areas of propodeum mainly rugulose-reticulate (almost entirely smooth in *N.subtropicalis*), areola of propodeum wide (narrow in *N.subtropicalis*), radial vein (r) of fore wing arising from middle of pterostigma (before middle in *N.subtropicalis*), second radiomedial vein (r-m) of the fore wing absent (present in *N.subtropicalis*), hind tibia distinctly thickened (rather slender in *N.subtropicalis*), basal area of second tergite not delineated by furrow (weakly delineated in *N.subtropicalis*), median length of second tergite (with apical area) 1.3× its basal width (almost equal in *N.subtropicalis*), ovipositor sheath not widened apically (distinctly widened in *N.subtropicalis*), and ovipositor sheath with sparse and long setae (with rather short and dense setae in *N.subtropicalis*).

*N.geochangus* sp. nov. is also similar to Neoheterospilus (N.) curvicaudis (Belokobylskij, 1994) from Vietnam, but it differs from the latter by the antenna 14-segmented and with the scape not compressed and long (20-segmented, with a weakly compressed and short scape in *N.curvicaudis*), penultimate segment of antenna 6.0× longer than wide and 1.1× longer than apical segment (4.0× longer than wide and 0.9× as long as the apical segment in *N.curvicaudis*), precoxal sulcus running along almost the entire length of the lower part of mesopleuron (along anterior 1/2 in *N.curvicaudis*), basolateral areas of propodeum mainly rugulose-reticulate (almost entirely smooth in *N.curvicaudis*), second radiomedial vein (r-m) of fore wing absent (present in *N.curvicaudis*), basal area of the second tergite absent (finely delineated by shallow furrow in *N.curvicaudis*), second suture of metasoma smooth (crenulate in *N.curvicaudis*), ovipositor sheath not widened apically (distinctly widened in *N.curvicaudis*), ovipositor sheath with sparse and long setae (with rather short and dense setae in *N.curvicaudis*), and pterostigma entirely pale brown (brown in *N.curvicaudis*).

##### Description.

**Female.** Body length 1.7 mm; fore wing length 1.3 mm.

***Head*.** Head width (dorsal view) 1.5× its median length. Occiput distinctly concave. Occipital carina mediodorsally straight, without medial break. Head behind eyes (dorsal view) distinctly and roundly narrowed. Transverse diameter of eye 1.5× longer than temple (dorsal view). POL 0.8× Od, 0.35× OOL. Eye 1.2× as high as broad. Malar space 0.35× eye height, 0.7× basal width of mandible. Face width 1.2× eye height and 1.5× height of face and clypeus combined. Hypoclypeal depression transverse and oval, its width 1.2× distance from edge of depression to eye, 0.5× width of face. Hypostomal flange narrow. Mandible medium size. Maxillary palpi almost as long as head height.

***Antenna*.** Antenna slender, filiform, 14-segmented, weakly shorter than body. Scape relatively long, not compressed, straight apically, with sparse white setae on inner side; length of scape almost 2.0× its maximum width, 1.4× longer than enlarged pedicel. First flagellar segment 6.5× longer than its apical width, 0.9× as long as second segment. Penultimate segment 6.0× longer than wide, almost as long as first segment, 1.1× longer than apical segment; the latter acuminate apically.

***Mesosoma*.** Mesosoma 1.9× longer than its height. Mesoscutum 0.7× as long as wide. Median lobe of mesoscutum almost straight anteriorly, with distinct subpointed lateral corners. Notauli distinct, rather deep, complete, and sparsely crenulate. Prescutellar depression with high median carina, smooth, 0.5× as long as weakly convex scutellum. Precoxal sulcus distinct, mainly crenulate, running along almost entire length of lower part of mesopleuron, but its posterior part visible as narrow stripe.

***Wings*.** Fore wing almost 3.0× longer than wide. Metacarp 1.3× longer than wide pterostigma. Second radiomedial vein (r-m) probably absent. First radial abscissa 0.8× as long as width of pterostigma, ~ 0.15× as long as second abscissa (3-SR + SR1), 0.4× as long as trace of first radiomedial vein (2-SR). Second abscissa (3-SR + SR1) distinctly evenly curved. First abscissa of medial vein (1-SR+M) distinctly curved. Recurrent vein (m-cu) postfurcal to trace of first radiomedial vein (2-SR). Discoidal (discal) cell 1.8× longer than wide. Nervulus (cu-a) short and weakly postfurcal, distance (1-CU1) between basal vein (1-M) and nervulus (cu-a) almost equal to nervulus (cu-a) length. Hind wing 6.0× longer than wide. Second costal abscissa (1-SC+R) mainly absent. First abscissa of mediocubital vein (M+CU) almost as long as second abscissa (1-M). Recurrent vein (m-cu) interstitial, unsclerotised, perpendicular to mediocubital vein (1-M).

**Figure 7.**
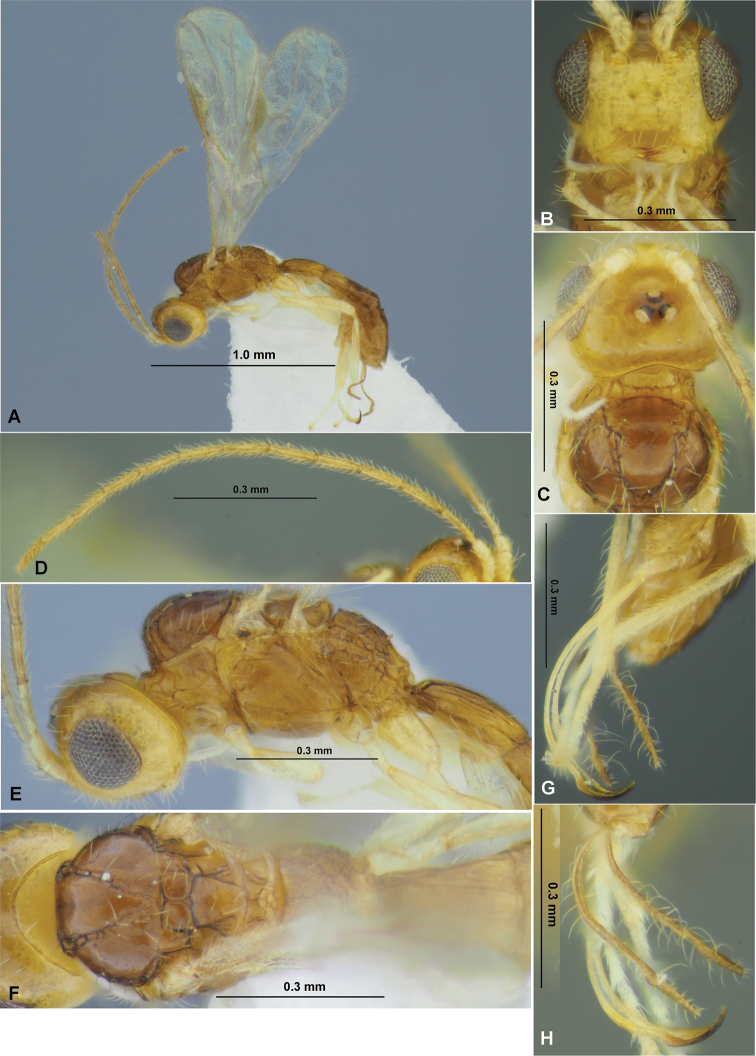
*Neoheterospilusgeochangus* sp. nov. (female, holotype) **A** habitus, lateral view **B** head, front view **C** head and mesoscutum, dorsal view **D** antenna **E** head and mesosoma, lateral view **F** mesosoma, dorsal view **G** ovipositor and sheaths, lateral view **H** ovipositor and sheaths, dorsal view.

**Figure 8.**
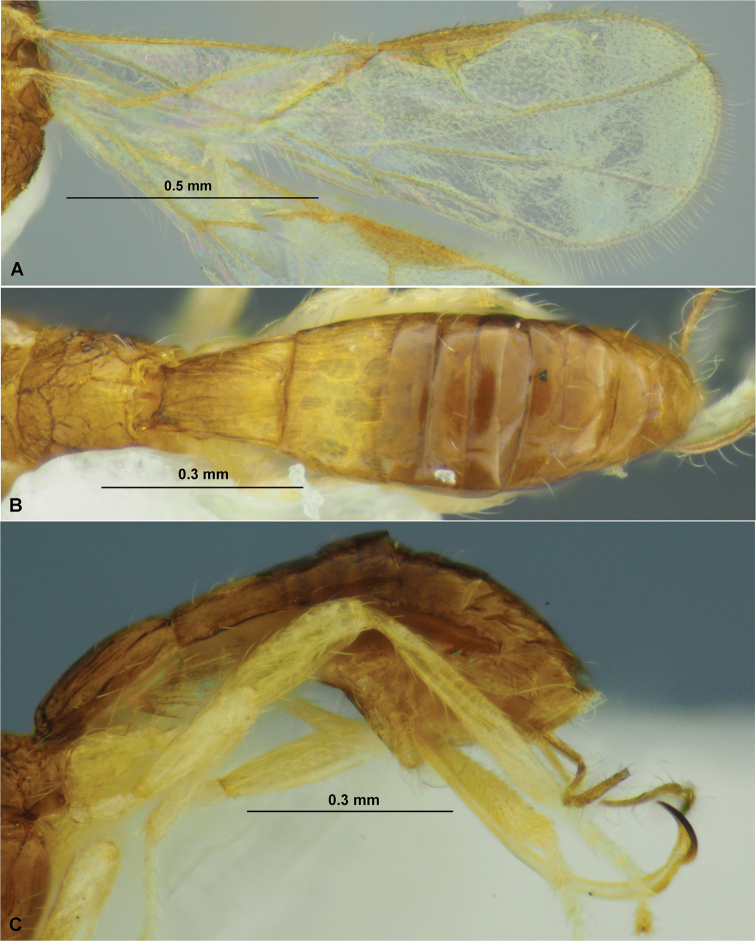
*Neoheterospilusgeochangus* sp. nov. (female, holotype) **A** wings **B** propodeum and metasoma, dorsal view **C** metasoma and ovipositor, lateral view.

***Legs*.** Hind femur 3.4× longer than wide. Hind tarsus 0.9× as long as hind tibia. Hind tibia distinctly thickened; hind tarsus thickened basally and narrowed distally. Hind basitarsus 0.4× as long as second–fifth segments combined. Second segment of hind tarsus 0.6× as long as basitarsus, almost as long as fifth segment (without pretarsus).

***Metasoma*.** Metasoma 1.2× longer than head and mesosoma combined. First tergite with weak spiracular tubercles in basal 1/3, weakly and linearly widened toward apex, its length 1.5× apical width; apical width almost 2.0× basal width. Basal area of second tergite not delineated by transverse furrow; apical area wide and delineated anteriorly by deep and almost straight crenulate furrow, medial length of this area 0.5× length of remaining tergite. Median length of second tergite (with apical area) 1.3× its basal width, approximately twice length of third tergite. Ovipositor sheath slender and not widened apically but with small ventral process in its subapical part; ~ 0.5× as long as metasoma, 0.8× as long as mesosoma, 0.3× as long as fore wing. Ovipositor slender and upcurved, its apex as on figures (Fig. 7G, H), with distinct subbasal ventral excise, its thickened apical part medium length.

***Sculpture and pubescence*.** Head entirely (including face) smooth. Mesoscutum mainly smooth, finely coriaceous anteriorly and along notauli at short areas, with weak convergent carinae in posterior 1/2. Scutellum and mesopleuron smooth at most part. Metapleuron entirely rugulose, finely sculptured anteriorly. Basolateral areas of propodeum short and wide, mainly rugulose-reticulate; remaining part of propodeum distinctly and rather densely rugose-reticulate and partly with transverse striation; areola more or less distinctly delineated by carinae, irregular shape, wide, approximately as long as wide; petiolate area delineated; basal carina situated in basal quarter. Hind coxa and femur smooth. First metasomal tergite distinctly, relatively sparsely and distally weakly curvedly longitudinally striate, with fine reticulation between striae, dorsal carinae distinct, complete, and convergent towards posterior margin. Second tergite distinctly and densely striate, but its apical area smooth. Remaining tergites smooth. Suture between second and third tergites smooth. Vertex almost entirely with sparse long and semi-erect white setae directed forwards. Mesoscutum mainly glabrous, with long, erect, and sparse white setae arranged narrowly along notauli and marginally. Mesopleuron glabrous in most part. Hind tibia with rather short, semi-erect and sparse white setae, their length 0.5–0.8× maximum width of tibia. Ovipositor sheath with sparse and long setae.

***Colour*.** Head brownish yellow to yellow in lower 1/2. Mesosoma pale reddish brown, pale anteriorly. Metasoma reddish brown to yellowish brown in anterior 0.4 and reddish brown in posterior 0.6. Antennae pale brown, yellow basally. Palpi pale yellow. Legs yellow to pale yellow, coxae infuscate in basal halves. Ovipositor sheath brown. Fore wing hyaline. Pterostigma entirely pale brown.

**Male.** Unknown.

##### Etymology.

Named after the type locality of the new species in South Korea, Geochang town.

##### Distribution.

Korean Peninsula.

#### ﻿Genus Spathius

Taxon classificationAnimaliaHymenopteraBraconidae

Nees, 1819

B33C82CC-A075-59A5-A3DC-E861880ABBD2

##### Type species.

*Cryptusclavatus* Panzer, 1809 (= *Ichneumonexarator* Linnaeus, 1758).

#### ﻿ Spathius fumipennis sp. nov.

Taxon classificationAnimaliaHymenopteraBraconidae

F2D0EEA3-FE04-5CA6-B31B-D6D219CAA959

https://zoobank.org/5A13443D-3137-44DD-87C8-7861DA6BD55B

Figs 9 , 10

##### Type material.

***Holotype***: female, “Korea, Kyongsangbuk-do [Gyeongsangbuk-do], Chomch’on-up [Jeomchon-eup], Daesong Buljong [Daeseong Buljeong], 9.VI.1992, D.-S. Ku” (NIBR).

***Paratype*.** 1 female, “Korea, Chungnam-do, Cheongyang-gun, Jeongsan-myeon, Machi-ri, sweeping, 15.VI.1992, D-S Ku” (SMNE).

##### Comparative diagnosis.

This new species belongs to the *S.fasciatus* Walker species group. *S.fuscipennis* sp. nov. is similar to Japanese *S.hikoensis* Belokobylskij, 1998 (Belokobylskij 1998), but differs from the latter species by having the occipital carina joined below with hypostomal carina (usually not joined and obliterated below in *S.hikoensis*); first flagellar segment 4.0× longer than its apical width (5.0–5.7× in *S.hikoensis*); the apical 1/2 of antenna completely dark and without pale subapical segments (with several pale subapical segments in *S.hikoensis*); the mesoscutum entirely weakly granulate-coriaceous (distinctly granulate in *S.hikoensis*); scutellum without lateral carinae (with distinct carinae in *S.hikoensis*); fore wing distinctly and evenly infuscate, pterostigma entirely brown (only faintly infuscate and pale in basal 1/3 in *S.hikoensis*); radial vein (r) arising distinctly behind middle of pterostigma, from its basal 2/3 (arising from basal 3/5 in *S.hikoensis*); hind femur weakly thicker, 4.1× longer than wide (slender, its length 4.3–4.8× longer than wide in *S.hikoensis*); setae on the dorsal surface of the hind tibia shorter, 0.7–1.0× as long as the maximum width of the tibia (long, 1.1–1.5× longer in *S.hikoensis*).

The new species is also similar to *S.clavator* Tang, Belokobylskij & Chen, 2015 (Tang et al. 2015) from China (Hainan), but differs from it by having the vertex almost entirely smooth (mainly rugulose-striate in *S.clavator*); malar space 0.6× eye height and almost equal to basal width of mandible (0.4× eye height and 0.7× basal width of mandible in *S.clavator*); occipital carina joined below with hypostomal carina (not joined and obliterated below in *S.clavator*); first flagellar segment 4.0× longer than its apical width (6.7× in *S.clavator*); mesoscutum entirely weakly granulate-coriaceous and without or with very short rugae (distinctly granulate and with long rugae in *S.clavator*); mesopleuron medially widely smooth, (entirely densely granulate with striation in *S.clavator*); hind femur slender, 4.1× longer than wide (thicker, its length 3.7× longer than wide in *S.clavator*); fore wing distinctly and evenly infuscate, pterostigma entirely brown (wing faintly infuscate, pterostigma pale in basal 1/3 in *S.clavator*); radial vein (r) of the fore wing arising distinctly behind the middle of the pterostigma, from its basal 2/3 (from middle in *S.clavator*); length of petiole 2.3× its apical width (2.7× in *S.clavator*); second tergite without separated laterotergites (with laterotergites separated in basal 1/2 in *S.clavator*).

##### Description.

**Female.** Body length 4.7–4.8 mm; fore wing length 3.2–3.4 mm.

***Head*.** Head width (dorsal view) 1.5× its median length, 1.2× width of mesoscutum. Head behind eyes (dorsal view) weakly convex in anterior 1/2 and roundly narrowed in posterior 1/2; transverse diameter of eye 1.2× length of temple. Ocelli medium-sized, arranged in triangle with base 1.1× its sides; POL 0.8× Od, 0.3× OOL. Eye glabrous, 1.2× as high as broad. Malar space 0.5–0.6× eye height and 1.0–1.3× basal width of mandible. Face width 1.3× eye height and 1.2–1.3× height of face and clypeus combined. Clypeal suture rather fine and complete. Clypeus weakly convex. Hypoclypeal depression transverse-oval, its width equal to distance from edge of depression to eye, 0.4–0.5× width of face. Occipital carina joined with hypostomal carina below upper base of mandible. Hypostomal flange wide and distinct. Vertex distinctly convex.

***Antenna*.** Antenna weakly thickened, almost setiform, 29–30-segmented, almost as long as body. Scape 1.8–1.9× longer than its width. First flagellar segment 4.0–4.5× longer than its apical width, 1.2–1.3× longer than second segment. Penultimate segment 2.0–2.5× longer than its wide, 0.4× as long as first flagellar segment, 0.8–0.9× as long as apical segment; the latter weakly acuminated apically.

***Mesosoma*.** Length of mesosoma 1.7× its height. Pronotal keel fine but distinct, its posterior branch medially widely fused with posterior margin of pronotum, anterior branch almost indistinct. Pronotum subanteriorly with rather distinct transverse carina. Pronotal lateral depression not delineated upper by carinae, wide, shallow, coarsely and rather densely curvedly crenulate. Mesoscutum highly and subvertically elevated above pronotum. Notauli complete, wide, rather deep anteriorly and more or less shallow posteriorly, densely, sparsely and distinctly crenulate with reticulation between rugae. Prescutellar depression rather deep, relatively long, with five almost complete carinae, finely rugulose or reticulate, 0.30–0.35× as long as scutellum. Scutellum weakly convex, without lateral carinae. Metanotum with short, wide and subpointed dorsal tooth. Subalar depression shallow, rather narrow, rugose-striate. Precoxal sulcus deep, wide, straight, oblique, distinctly crenulate, with fine reticulation, running along anterior 0.6 of lower length of mesopleuron, with shallow and relatively wide striate depression behind sulcus. Metapleural flange wide and short. Propodeum with distinct, short, and thick lateral tubercles.

***Wings*.** Fore wing 3.6–3.8× longer than wide. Pterostigma 4.2–5.0× longer than its maximum width. Radial vein (r) arising distinctly behind middle of pterostigma, inner distance of pterostigma from parastigma to radial vein (r) 1.7–1.8× its inner distance from radial vein to metacarp (1-R1). Radial (marginal) cell not shortened; metacarp (1-R1) 1.4× longer than pterostigma. First radial abscissa (r) 0.8–0.9× as long as maximum width of pterostigma. Second radial abscissa (3-SR) 2.8–3.0× longer than first abscissa and forming with it very obtuse angle, 0.4–0.5× as long as straight third abscissa (SR1), 0.9× as long as first radiomedial vein (2-SR). Second radiomedial (submarginal) cell not or only weakly narrowed distally, its length 2.7× maximum width, 1.3× length of brachial (subdiscal) cell. First medial abscissa (1-SR+M) straight or weakly sinuate. Recurrent vein (m-cu) weakly postfurcal, ~ 6.0× longer than second abscissa of medial vein (2-SR+M), 0.4× as long as first radiomedial vein (2-SR). Nervulus (cu-a) weakly postfurcal, distance (1-CU1) from nervulus (cu-a) to basal vein (1-M) 0.3–0.4× nervulus (cu-a) length. Parallel vein (CU1a) not interstitial, arising from anterior 0.3–0.4 of distal vein (3-CU1) of brachial (subdiscal) cell. Mediocubital vein (M+CU1) almost straight or weakly curved. Hind wing 4.5–5.0× longer than maximum width. First costal abscissa (C+SC+R) 0.6× as long as second abscissa (1-SC+R). First abscissa of mediocubital vein (M+CU) 0.70–0.75× as long as second abscissa (1-M). Recurrent vein (m-cu) not pigmented, transparent, rather long, weakly antefurcal, distinctly oblique towards base of wing.

***Legs*.** Fore tibia with slender numerous and rather sparse spines arranged in single vertical line. Hind coxa 1.6× longer than its maximum width, with basoventral corner and small tooth. Hind femur claviform, 4.0–4.1× longer than wide. Hind tarsus 0.9–1.0× as long as hind tibia. Hind basitarsus 0.70–0.75× as long as second–fifth segments combined; second segment 0.45–0.50× as long as basitarsus, 1.1–1.3× longer than fifth segment (without pretarsus).

***Metasoma*.** Petiole (lateral view) weakly and evenly curved ventrally, dorsally distinctly and regularly curved to its middle and almost straight in apical 1/2, thickened submedially; widened on spiracle level and weakly widened in apical 0.2–0.3 (dorsal view), with small spiracular tubercles in basal 1/3. Length of petiole 2.3–2.4× its apical width, almost 2.0× length of propodeum; apical width ~ 1.5× its width at level of spiracles. Second and following tergites without separate laterotergites. Second suture absent. Median length of second and third tergites combined 1.7–1.8× basal width of second tergite, 0.8× their maximum width. Ovipositor weakly curved down. Ovipositor sheath 1.3–1.4× longer than metasoma, 2.2–2.4× longer than mesosoma, 1.0–1.1× longer than fore wing.

***Sculpture and pubescence*.** Vertex almost entirely smooth, only finely coriaceous near ocelli; frons almost entirely with distinct, dense, and curved transverse striae, with additional fine reticulation between striae. Face entirely or mainly (in upper 2/3) densely and coarsely striate, with rugulosity between striae below and laterally, finely reticulate-coriaceous to smooth in lower lateral 1/3. Temple entirely smooth. Mesoscutum entirely densely and weakly granulate-coriaceous, sometimes with short rugae near notauli and laterally, coarsely, and sparsely rugose in wide and short medioposterior area. Scutellum densely and finely to very finely coriaceous, with fine transverse aciculae anteriorly. Mesopleuron medially widely almost smooth, finely and densely rugulose-reticulate marginally. Propodeum with areas delineated by distinct carinae; basolateral areas entirely and densely granulate-rugulose; areola wide and rather long, transverse striae with rugulosity, almost as long as wide; petiolate area rather long and wide, distinctly separated from areola by curved carina; basal carina 0.7–1.0× as long as anterior fork of areola. Hind coxa dorsally partly densely transversely striate with dense rugosity in wide basal 1/2, laterally distinctly and densely rugulose-granulate. Hind femur mainly smooth, longitudinally striate dorsally. Petiole distinctly and sparsely striate, with dense to very dense rugulosity between striae, only densely rugose in basal 1/4. Second and following tergites entirely smooth. Vertex with sparse, short, and almost erect pale setae situated laterally and anteriorly, glabrous on wide medial part. Mesoscutum with sparse, long, and erect yellow setae laterally and along notauli, glabrous on wide medial parts of lobes. Mesopleuron widely glabrous. Setae of dorsal surface of hind tibia erect, rather dense, mainly long, their length 0.7–1.0× maximum width of tibia.

**Figure 9.**
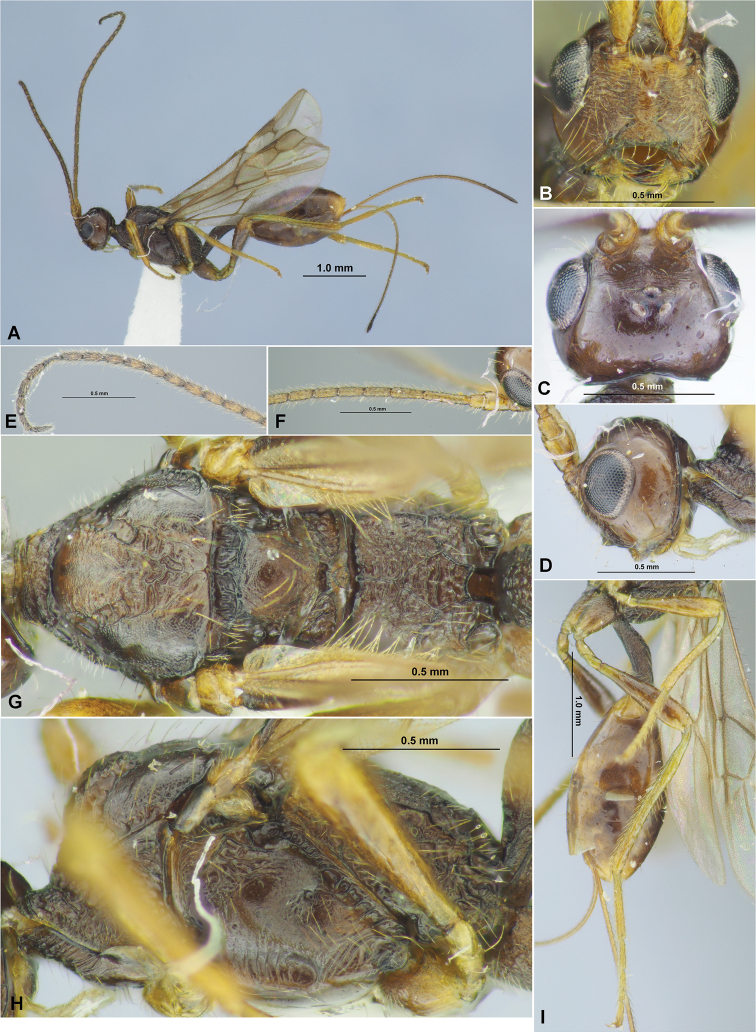
*Spathiusfumipennis* sp. nov. (female, holotype) **A** habitus, lateral view **B** head, front view **C** head, dorsal view **D** head, lateral view **E** apical segments of antenna **F** basal segments of antenna **G** mesosoma, dorsal view **H** mesosoma, lateral view **I** hind leg.

**Figure 10.**
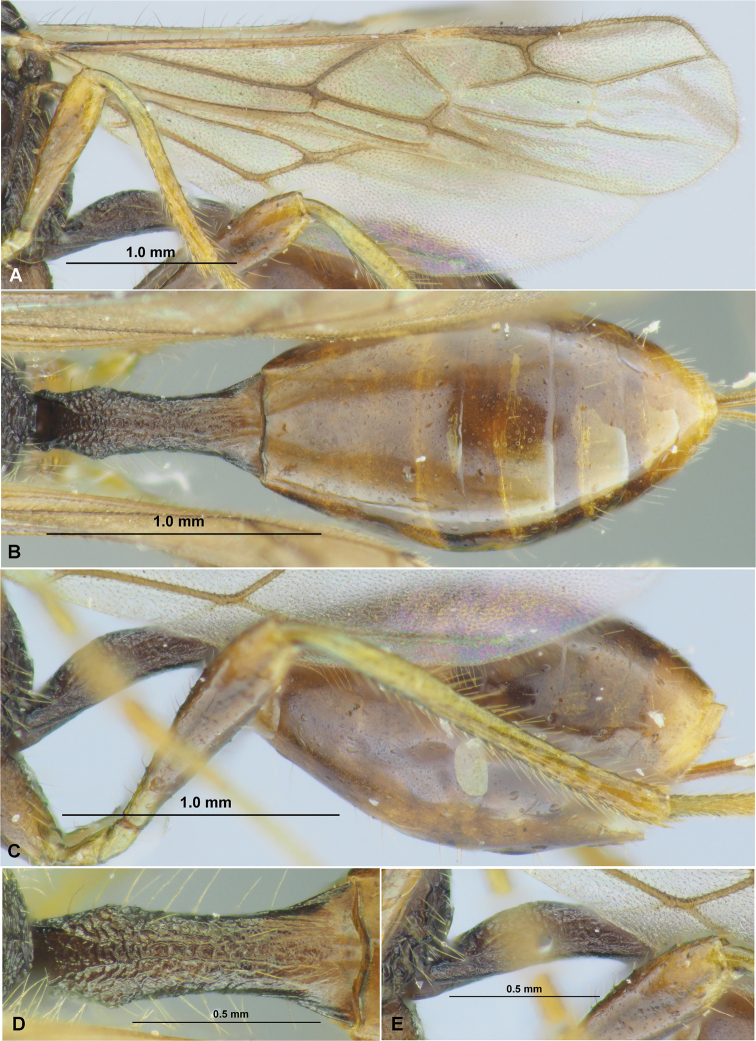
*Spathiusfumipennis* sp. nov. (female, holotype) **A** wings **B** metasoma, dorsal view **C** metasoma, lateral view **D** petiole, dorsal view **E** petiole, lateral view.

***Colour*.** Body mainly dark reddish brown to almost black partly, metasoma posteriorly or already behind petiole and ventrally reddish brown. Antennae brown with dark brown apical quarter or mainly dark brown with two basal segments pale brown, without pale subapical segments. Palpi yellow or brownish yellow. Legs partly reddish brown or pale reddish brown, all trochanters and trochantelli, tibiae and tarsi yellow or yellowish brown, hind tibiae basally yellow on rather long distance. Ovipositor sheath mainly pale brown, almost black apically. Fore wing distinctly and evenly infuscate, faintly paler basally and apically. Pterostigma evenly brown or yellowish brown in basal third.

**Male.** Unknown.

##### Etymology.

This species is named from the Latin *fumis* (= smoke) and *pennis* (= pen, “wing”), after its distinctly infuscate fore wing.

##### Distribution.

Korean Peninsula.

###### New and rare species in the fauna of Korean Peninsula

#### ﻿* Dendrosoter middendorffii

Taxon classificationAnimaliaHymenopteraBraconidae

(Ratzeburg, 1848)

0CF4FD97-9D39-5F12-97B0-5EC5FF6D1815

Bracon (Eurybolus) middendorffii Ratzeburg, 1848: 32.
Dendrosoter middendorffii : Belokobylskij and Tobias 1986: 39; Belokobylskij 1996: 66; Belokobylskij and Maetô 2009: 93; Yu et al. 2016; Belokobylskij et al. 2019: 261.

##### Material examined.

South Korea: 1 female, “S. Korea, Gyeongsangnam-do (GN), Geochang-gun, Namsang-myeon, Jeoncheok-ri, 35°37'23.1"N, 127°56'31.8"E, 11.06.2022, Tselikh, Lee, Belokobylskij”, “Reared from the logs of *Pinusdensiflora* infested by Curculionidae (Scolytinae) by 13.06.2022” (NIBR); 2 females, same locality and data (ZISP); 1 female, same locality and data, but emerged 29.VI.2022 (SMNE); 1 female, same locality and data, but emerged 2.VII.2022 (SMNE).

##### Distribution.

*Korean Peninsula; Europe, Georgia, Armenia, Turkey, Israel, Iran, Russia (European part, Urals, south of Far East), Japan, India.

#### ﻿ Eodendrus eous

Taxon classificationAnimaliaHymenopteraBraconidae

(Belokobylskij, 1988)

67687965-54FA-52F6-8533-530DAEEF8549

Dendrosotinus eous Belokobylskij, 1988: 625.
Dendrosotinus (Eodendrus) eous : Belokobylskij 1998: 66.
Eodendrus eous : Belokobylskij et al. 2005: 2731; Belokobylskij and Maetô 2009: 153; Yu et al. 2016; Lee et al. 2020: 18.

##### Material examined.

South Korea: 1 female, SW of Geochang-eup, forest on mountain, 35°40'15"N, 127°53'24"E – 35°40'19"N, 127°53'01"E, 20.06.2019, K. Samartsev (ZISP); 1 male, SW of Geochang-eup, forest on a mountain, 35°40'15"N, 127°53'24"E – 35°40'19"N, 127°53'01"E, 3.06.2019, K. Samartsev (ZISP); 1 female, “Korea (GB), Cheonbu3-gil, Buk-myeon, Ulleung-gun, VIII.15–VIII.31.2017 (Malaise trap), Ku Deokseo” (SMNE); 1 female, “Korea (GB), Hakpo-ri, Seo-myeon, Ulleung-gun, VIII.15–VIII.31.2017 (Malaise trap), Ku Deokseo” (SMNE); 1 female, “Korea: CN, Keumsan, Kunbuk, Sanan, Jajinbaengii, 19–24.V.1998, MT, Tripotin Pierre” (SMNE); 1 female, “Korea: GG, Suwon, Mt. Yeogi, Matsumura, 23.VI.1997, June-Yeol Choi” (SMNE).

##### Distribution.

Korean Peninsula; Russia (south of the Far East), Japan.

#### ﻿* Guaygata mariae

Taxon classificationAnimaliaHymenopteraBraconidae

(Belokobylskij, 1993)

0D5EF777-8CDB-50AF-BFBF-64BFB8182016

Neurocrassus mariae Belokobylskij, 1993: 163.
Guaygata mariae : Belokobylskij and Maetô 2006: 604; 2009: 160; Tang et al. 2013: 85; Yu et al. 2016.

##### Material examined.

South Korea: 1 female, “Korea (GB), Cheonbu3-gil, Buk-myeon, Ulleung-gun, X.3–XI.14.2017 (Malaise trap), Ku Deokseo” (NIBR); 1 female, “Korea. Konbongsa, Kosong, Kangwon. 26.V.1993. Deok-Seo Ku” (SMNE).

##### Distribution.

*Korean Peninsula; Russia (Primorskiy Territory), China (Jiangsu, Fujian); Japan (Honshu), Vietnam.

#### ﻿* Ipodoryctes signipennis

Taxon classificationAnimaliaHymenopteraBraconidae

(Walker, 1860)

0532781B-F38B-5DC3-B77F-9466DABA960F

Spathius signipennis Walker, 1860: 309.
Rhaconotus signipennis : Belokobylskij 2001: 134.
Ipodoryctes signipennis : Belokobylskij and Zaldívar-Riverón 2021: 44.

##### Material examined.

South Korea: 1 female, “Korea [JN], Jeungdo-myeon, Daecho-ri, Sinan-gun, 35°58'56"N, 126°9'23"E, 2020.VIII.31–IX.15, Coll. S.W. Choi & J.Y. Lee, The 5^th^ National Ecosystem Survey” (NIBR).

##### Distribution.

*Korean Peninsula; Russia (Far East), China, Japan, India, Sri Lanka, Vietnam, Indonesia.

#### ﻿* Leluthia (Leluthia) disrupta

Taxon classificationAnimaliaHymenopteraBraconidae

(Belokobylskij, 1994)

80B47084-FA86-59E3-97C3-F75A8ABA811B

Pareucoryctes disruptus Belokobylskij, 1994: 23.
Leluthia (Leluthia) disrupta : Belokobylskij and Maetô 2009: 294; Li et al. 2015: 595; Yu et al. 2016.

##### Material examined.

South Korea: 1 female, “Korea, Gangwon-do, Inje, Sangnam, Misan-ri, Wangseong-dong, Mt. Bangtaesan, 12.VIII.1986. Deok-Seo Ku” (NIBR).

##### Distribution.

*Korean Peninsula; Russia (North Caucasus, south of Far East), Georgia.

#### ﻿ Leluthia (Leluthia) honshuensis

Taxon classificationAnimaliaHymenopteraBraconidae

Belokobylskij & Maetô, 2006

7E73C46A-8371-524A-A2D7-B976E258F921

Leluthia (Leluthia) honshuensis Belokobylskij & Maetô, 2006: 607; 2009: 295; Yu et al. 2016; Kim et al. 2018: 135.

##### Material examined.

South Korea: 1 female, “Korea, Gyeongnam-do, Hadong-gun, Bukcheon-myeon, Jikjeon-ri, Mt. Limyeong (Light trap), 28–29.VIII.2000, J-S Park” (SMNE).

##### Distribution.

Korean Peninsula (Kim et al. 2018); Japan.

#### ﻿* Leluthia (Leluthia) nagoyae

Taxon classificationAnimaliaHymenopteraBraconidae

Belokobylskij & Maetô, 2006

C3FD82A4-DBCE-52F1-8527-1D32CCBF073F

Leluthia (Leluthia) nagoyae Belokobylskij & Maetô, 2006: 610; 2009: 299; Yu et al. 2016.

##### Material examined.

South Korea: 1 male, “Korea (GN), Geochang-gun, Geochang-eup, Science Museum Natural Enemy, IX.4–XI.16.2020 (Malaise Trap), Ku Deokseo” (NIBR).

##### Distribution.

*Korean Peninsula; Japan.

#### ﻿* Leluthia (Euhecabolodes) transcaucasica

Taxon classificationAnimaliaHymenopteraBraconidae

(Tobias, 1976)

AF4D080B-5D7A-54AB-A4E1-F57735399052

Euhecabolodes transcaucasicus Tobias, 1976: 251.
Leluthia (Euhecabolodes) transcaucasica : Belokobylskij and Maetô 2009: 294; Li et al. 2015: 595; Yu et al. 2016.

##### Material examined.

South Korea: 1 female, “Korea, Gyeonggi-do, Osan, Sucheong-dong, Gyeonggi-do, Forest Environment Research Institute (Light trap), 1.VI.1999, H-G Lee” (NIBR); 1 female, “Korea, Chungbuk-do, Danyang-gun, Danyang-eup, Cheongdong-ri, Cheondong valley, Mt. Sobaek, 14.VIII.1998. J-S Park” (SMNE).

##### Distribution.

*Korean Peninsula; Czech Republic, Russia (Buryatia, south of the Far East), Georgia, Turkey, Iran, Kazakhstan, Mongolia.

#### ﻿* Neoheterospilus (Neoheterospilus) subtropicalis

Taxon classificationAnimaliaHymenopteraBraconidae

Belokobylskij, 1996

B4360383-3983-5A73-AB2F-D2F76F8031F3

Neoheterospilus (Neoheterospilus) subtropicalis Belokobylskij, 2006: 173; Belokobylskij and Maetô 2009: 316; Yu et al. 2016.

##### Material examined.

South Korea: 1 female, “S. Korea: Gyeongsangnam-do, Goseong-gun, Hail-myeon, Suyang-ri, 34°58'08"N, 128°12'08.3"E, 18.VI.2022, Tselikh col.” (NIBR).

##### Distribution.

*Korean Peninsula; China, Japan, Vietnam.

#### ﻿* Pareucorystes varinervis

Taxon classificationAnimaliaHymenopteraBraconidae

Tobias, 1961

D1ADC0F4-1CFE-5068-AD12-65E22AB66B6F

Pareucorystes varinervis Tobias, 1961: 533; Yu et al. 2016; Belokobylskij 2019: 37.
Leluthia chinensis Li & van Achterberg, in Li et al. 2015: 595; Belokobylskij 2019: 37 (as synonym).

##### Material examined.

South Korea: 1 female, “Gyeongnam-do, Namhae I., Mt. Geumsan, 2017.7.21–22 (LT), Deokseo Ku, Taeho Ahn, Hyerin Lee coll.” (NIBR).

##### Distribution.

*Korean Peninsula; Europe, Russia (European part, south of Far East), Azerbaijan, Kazakhstan, China.

#### ﻿* Rhaconotinus (Rhaconotinus) tianmushanus

Taxon classificationAnimaliaHymenopteraBraconidae

(Belokobylskij & Chen, 2004)

C1E9B689-EB3F-5621-8AF0-B604C25459F2

Rhaconotus tianmushanus Belokobylskij & Chen, 2004: 349 (Rhaconotus); Yu et al. 2016.
Rhaconotinus (Rhaconotinus) tianmushanus : Belokobylskij and Zaldívar-Riverón 2021: 109.

##### Material examined.

South Korea: 1 female, “S. Korea [GB], Changyeong-gun, Yueo-myeon, Daedae-ri, Uponeup, 3.VII.2015, Tselikh” (NIBR).

##### Distribution.

*Korean Peninsula; China (Zhejiang).

#### ﻿* Spathius deplanatus

Taxon classificationAnimaliaHymenopteraBraconidae

Chao, 1978

AAB7CB98-0D78-57FF-917C-0A9DDC85F748

Spathius deplanatus Chao, 1978: 180; Belokobylskij 2003: 485; Chen and Shi 2004: 128; Belokobylskij and Maetô 2009: 558; Tang et al. 2015: 40; Yu et al. 2016.

##### Material examined.

South Korea: 1 female, “Korea: KK, Suwon, Mt. Yeogi, MT (B1/B1), 23.IX.1998, June-Yeol Choi” (NIBR); 1 female, “Korea: KK, Suwon, Mt. Yeogi, MT (Wh/Gr), 28.VII.1997, June-Yeol Choi” (SMNE);1 female, “Korea (GN), Geochang-gun, Science Museum Natural Enemy, XI.8–XI.23.2021 (Malaise Trap), Ku Deokseo, Lee Jaehyeon” (SMNE).

##### Distribution.

*Korean Peninsula; China, Japan.

#### ﻿* Spathius honshuensis

Taxon classificationAnimaliaHymenopteraBraconidae

Belokobylskij, 1998

1ED53E51-1484-5BD2-9A75-18D372DD123D

Spathius honshuensis Belokobylskij, 1998: 83; 2003: 469; Belokobylskij and Maetô 2009: 599; Yu et al. 2016.

##### Material examined.

South Korea: 1 male, “Korea: Seoul, Hongneung, National Institute of Forest Science, Light trap, 3.IX.1998, Kang Seung-Ho” (NIBR).

##### Distribution.

*Korean Peninsula; Japan.

#### ﻿* Spathius longipetiolus

Taxon classificationAnimaliaHymenopteraBraconidae

Belokobylskij & Maetô, 2009

E8BB91DD-DC3A-5D0A-823A-E8D55E5B6DC1

Spathius longipetiolus Belokobylskij & Maetô, 2009: 631; Tang et al. 2015: 60; Yu et al. 2016.

##### Material examined.

South Korea: 1 female, “Korea (GN), Geochang-gun, Science Museum Natural Enemy, VI.19–VII.19.2014 (Malaise Trap), Ku Deokseo” (NIBR); 1 female, same locality, but VIII.16–IX.4.2020 (SMNE); 2 females, “Korea (GN), Ungseokbong, Nae-ri, Sancheong-gun, VI.17.2017 (Sweeping), Ahn Taeho” (SMNE, ZISP); 1 female, “Korea (GB), Cheonbu3-gil, Buk-myeon, Ulleung-gun, VIII.16–VIII.30.2017 (Malaise trap), Ku Deokseo” (SMNE); 1 female, same label, but VIII.2–VIII.16.2017 (SMNE); 1 female, same label, but IX.10–IX.13.2017 (ZISP).

##### Distribution.

*Korean Peninsula; China, Japan.

#### ﻿* Spathius pseudaspersus

Taxon classificationAnimaliaHymenopteraBraconidae

Belokobylskij, 2009

2264EEF8-F971-5939-B188-0DEDD7C19430

Spathius pseudaspersus Belokobylskij, 2009: 455; Belokobylskij and Maetô 2009: 691; Tang et al. 2015: 91.

##### Material examined.

South Korea: 2 females, “Korea (GN), Ungseokbong, Nae-ri, Sancheong-gun, VI.17.2017 (Sweeping), Ahn Taeho” (NIBR, SMNE); 1 female, “Korea (JN), Jangjoa-ri, Wando-eup, Wando-gun, IX.26–X.10.2020 (Malaise Trap), Ku Deokseo, Lee Jaehyeon” (SMNE); 1 female, “S. Korea [GB], Changyeong-gun, Irwol-myeon, Mt. Ilwol-san, 36°48'29"N, 129°05'25"E, 25.VII.2015, Tselikh” (ZISP).

##### Distribution.

*Korean Peninsula; Russia (Far East), China (Jiangsu), Japan.

#### ﻿* Spathius sinicus

Taxon classificationAnimaliaHymenopteraBraconidae

Chao, 1957

7842F090-B632-57C1-98A7-7BF2CD047A4B

Spathius sinicus Chao, 1957: 3; 1977: 209; Chen and Shi 2004: 162; Tang et al. 2015: 106; Yu et al. 2016.
Spathius bellus Chao, 1957: 5; Chen and Shi 2004: 112; Tang et al. 2015: 106 (as synonym).
Spathius agrili Yang, in Yang et al. 2005: 638; Belokobylskij and Maetô 2009: 510; Tang et al. 2015: 106 (as synonym).

##### Material examined.

South Korea: 1 female, “Korea: KK, Suwon, Mt. Yeogi, MT (B1/B1), 23.IX.1996, June-Yeol Choi” (NIBR); 1 female, same label, but 9.VI.1997 (SMNE); 1 female, “Korea: Kuonggi, Kwangju, Docheok, Sangrim, Mt. Taewha, 5.VIII.1998, Deok-Seo Ku (LT)” (SMNE); 1 female, “Gyeongbuk, Uiseong-gun, Gaeum-myeon, Hyunri-ri, Yongsan, Mt. Seonamsan, 9.V.1998. T-H Ahn (Sweeping)” (SMNE); 1 female, “Gyeongnam, Hadong-gun, Cheongam-myeon, Gunghangk-ri, Jusan, 1.VII.2002 (Sweeping), J-S Park” (SMNE); 1 female, ”S. Korea: Gyeongsangnam-do, Sancheong-gun, 30 km NNW Jinju (Chinju), forest, bush, h = 800 m, 12.06.2002, S. Belokobylskij” (ZISP); 1 female, “South Korea, SW of Geochang-eup, forest on a mountain, 35°40'15"N, 127°53'24"E – 35°40'19"N, 127°53'01"E, 3.VI.2019, K. Samartsev (ZISP).

##### Distribution.

*Korean Peninsula; China, Japan.

#### ﻿* Spathius tsukubaensis

Taxon classificationAnimaliaHymenopteraBraconidae

Belokobylskij & Maetô, 2009

DA0EF10C-0D28-56B3-BAAA-0D141ADD480A

Spathius tsukubaensis Belokobylskij & Maetô, 2009: 730; Yu et al. 2016.

##### Material examined.

South Korea: 1 female, “Korea, Gyeongbuk-do, Sangju-gun, Chupungryeong, VIII. 1989. D-S Ku” (NIBR); 1 female, “Korea: KK, Suwon, Mt. Yeogi, MT (B1/B1), 31.VII.1995, June-Yeol Choi” (SMNE); 1 female, “Korea (GB), Cheonbu3-gil, Buk-myeon, Ulleung-gun, VI.21–VII.5.2017 (Malaise trap), Ku Deokseo” (SMNE); 1 female, “Korea (JJ), Hala-Arboretum, Yeon-dong, Jeju-si, Jeju-do, VI.25–VII.09.2017, (Malaise Trap)” (SMNE).

##### Distribution.

*Korean Peninsula; Japan.

**Subfamily Rhyssalinae Foerster, 1863**

###### Tribe Acrisidini Hellén, 1957

#### ﻿Genus Acrisis

Taxon classificationAnimaliaHymenopteraBraconidae

Foerster, 1863

5F99B11B-6E06-52B7-9805-7D8F9002E3F3

##### Type species.

*Acrisisgracilicornis* Foerster, 1863.

#### ﻿ Acrisis brevicornis

Taxon classificationAnimaliaHymenopteraBraconidae

Hellén, 1957

E82E3FDA-20D2-55A2-9DF9-3B5CFB7BC64E

Acrisis brevicornis Hellén, 1957 (female): 50; Tobias 1983: 165; Belokobylskij 1990: 36; 1998: 120; Yu et al. 2016.
Acrisis koponeni Tobias, 1983 (male): 163; Belokobylskij 1990: 36 (as synonym); 1998: 120; Yu et al. 2016.

##### Material examined.

South Korea: 1 female, “Korea (GN), Geochang-gun, Science Museum Natural Enemy, VIII.23–IX.21.2014 (Malaise Trap), Ku Deokseo” (SMNE); 1 female, same label, but VIII.27–IX.21.2014 (SMNE); 1 female, same label, but VI.19–VII.19. 2014 (ZISP); 8 females, same label, but I. 1–VI.18.2015 (SMNE, ZISP); 2 females, same label, but VIII.8–IX.22.2015 (SMNE, ZISP); 1 female, same label, but IV.29–V.31.2017 (SMNE); 1 female, same label, but IX.4–IX.16.2020 (SMNE); 1 female, “Korea (GN), Geochang-gun, Science Museum Natural Enemy, VI.30–VII.14.2021 (Malaise Trap), Ku Deokseo, Lee Jaehyeon (SMNE); 1 female, same label, but IV.23–V.7.2022 (SMNE); 4 females, same label, but VI.4–VI.18.2022 (SMNE, ZISP); 2 females, “Korea (GN), Janggi-ri, Wicheon-myeon, Geochang-gun, IX.11–X.16.2015 (Malaise Trap), Ahn Taeho” (SMNE, ZISP); 1 male, same locality, but IV.1–V.17.2017; 1 female, “S. Korea [GB], Gumi-shi, Goa-eup, Goepyeong-ri, 944–91, 36°15'69"N, 128°37'75"E, 23.VI.2015, Tselikh” (ZISP); 1 female, “Korea (CB), Sanoe, Sinjeong, Boeun, 36°34'8.93"N, 127°48'35.52"E, 2020.VI.07–24, Coll. H.K. Lee & M.D. Yun, The 5^th^ National Ecosystem Survey” (SMNE).

##### Distribution.

Korean Peninsula; Spain, Hungary, Finland, Iran, Russia (north and north-west of the European part, south of Far East).

#### ﻿Genus Proacrisis

Taxon classificationAnimaliaHymenopteraBraconidae

Tobias, 1983

6558AC13-BA54-5F0B-9AAE-FD3112675F96

##### Type species.

*Proacrisisrarus* Tobias, 1983.

#### ﻿* Proacrisis orientalis

Taxon classificationAnimaliaHymenopteraBraconidae

Tobias, 1983

C1F39AFD-46DB-5145-B5A2-D557F2446E0D

Proacrisis orientalis Tobias, 1983: 162 (female); Belokobylskij 1994: 74; 1998: 120; Yu et al. 2016.
Proacrisis striatus Tobias, 1983: 161 (male); Belokobylskij 1994: 74 (synonym); 1998: 120; Yu et al. 2016.

##### Material examined.

South Korea: 1 female, “Korea [GW], Bangdon-ri, Girin-myeon, Inje-gun, VI.21–VII.29.2019 (Malaise Trap), HDS” (NIBR).

##### Distribution.

*Korean Peninsula; Russia (Far East).

###### Tribe Histeromerini Fahringer, 1930

#### ﻿Genus Histeromerus

Taxon classificationAnimaliaHymenopteraBraconidae

Wesmael, 1838

86EC0B3D-F4C2-568B-8744-5E84FC16B86D

##### Type species.

*Histeromerusmystacinus* Wesmael, 1838.

#### ﻿* Histeromerus orientalis

Taxon classificationAnimaliaHymenopteraBraconidae

Chou & Chou, 1991

B4E80A93-BF4D-5BD0-B722-DCE709277E85

Histeromerus orientalis Chou & Chou, 1991: 473; van Achterberg 1992: 195; Maetô 1997: 440; Belokobylskij 1998: 110; Yu et al. 2016.

##### Material examined.

South Korea: 1 female, “Korea [JJ], Eoseungsaengak, Haeando, Jeju-shi, Jejudo, V.15–V.28.2017, (Malaise Trap)” (NIBR).

##### Distribution.

*Korean Peninsula; China (Taiwan), Japan (Ogasawara).

## ﻿Discussion

The subfamily Doryctinae is rather well studied group of parasitoids in several East Asian countries. For example, on the basis of the last revision of the Japanese Doryctinae (Belokobylskij and Maetô 2009) totally 33 genera of this subfamily were recorded in the fauna of this Archipelago, including such rare for the Palaearctic region taxa as *Arhaconotus* Belokobylskij, 2001, *Asiaheterospilus* Belokobylskij & Konishi, 2001, *Cryptontsira* Belokobylskij, 2008, *Mimipodoryctes* Belokobylskij, 2001, *Nipponecphylus* Belokobylskij & Konishi, 2001, *Rhacontsira* Belokobylskij, 1998, *Ryukyuspathius* Belokobylskij, 2008, and *Spathiostenus* Belokobylskij, 1993. This subfamily is also abundant in China (32 genera) and rather similar to the Japanese fauna in its composition., which includes Palaearctic and Oriental elements. Already 26 doryctine genera were recorded in the fauna of Korean Peninsula, but this is clearly not complete information for the region. For example, at least some genera recorded already in Japan (*Caenophanes* Foerster, 1863, *Cryptontsira*, *Ecphylus* Foerster, 1863, *Mimipodoryctes*, *Parallorhogas* Marsh, 1993, and *Rhacontsira*) could be additionally found in the fauna of this peninsula. The less varied doryctine taxa are recorded in the Russian Far East, the northernmost Asian territory: only 20 genera were found here (Belokobylskij et al. 2019) and basically without any Oriental components in its composition.

The information about the discovery of the genera subfamily Rhyssalinae in the discussed region was usually much reduced. Such, only two rhyssaline genera (out of 11 worldwide known), *Lysitermoides* van Achterberg, 1995 and *Oncophanes* Foerster, 1863, were recorded in the fauna of Japan, and only single genus *Histeromerus* Wesmael, 1838 were found in China for now. On the other hand, already seven rhyssaline genera were recorded in the faunas of the Russian Far East and Korean Peninsula, but if in the first area the genera *Histeromerus* and *Tobiason* Belokobylskij, 2004 were not found till now, then on the latter territory the genera *Pseudobathystomus* Belokobylskij, 1986 and *Rhyssalus* Haliday, 1833 were not discovered yet; however both areas have five identical genera, namely *Acrisis* Foerster, 1863, *Proacrisis* Tobias, 1983, *Dolopsidea* Hincks, 1944, *Lysitermoides* van Achterberg, 1995, and *Oncophanes* Foerster, 1863.

## Supplementary Material

XML Treatment for Aivalykus

XML Treatment for Aivalykus kseniae

XML Treatment for Dendrosotinus

XML Treatment for Dendrosotinus (Gildoria) gajwadongus

XML Treatment for Doryctes

XML Treatment for Doryctes (Plyctes) jinjuensis

XML Treatment for Neoheterospilus

XML Treatment for Neoheterospilus (Neoheterospilus) geochangus

XML Treatment for Spathius

XML Treatment for Spathius fumipennis

XML Treatment for Dendrosoter middendorffii

XML Treatment for Eodendrus eous

XML Treatment for Guaygata mariae

XML Treatment for Ipodoryctes signipennis

XML Treatment for Leluthia (Leluthia) disrupta

XML Treatment for Leluthia (Leluthia) honshuensis

XML Treatment for Leluthia (Leluthia) nagoyae

XML Treatment for Leluthia (Euhecabolodes) transcaucasica

XML Treatment for Neoheterospilus (Neoheterospilus) subtropicalis

XML Treatment for Pareucorystes varinervis

XML Treatment for Rhaconotinus (Rhaconotinus) tianmushanus

XML Treatment for Spathius deplanatus

XML Treatment for Spathius honshuensis

XML Treatment for Spathius longipetiolus

XML Treatment for Spathius pseudaspersus

XML Treatment for Spathius sinicus

XML Treatment for Spathius tsukubaensis

XML Treatment for Acrisis

XML Treatment for Acrisis brevicornis

XML Treatment for Proacrisis

XML Treatment for Proacrisis orientalis

XML Treatment for Histeromerus

XML Treatment for Histeromerus orientalis
